# Inhibition of histone methyltransferase EZH2 in *Schistosoma mansoni in vitro* by GSK343 reduces egg laying and decreases the expression of genes implicated in DNA replication and noncoding RNA metabolism

**DOI:** 10.1371/journal.pntd.0006873

**Published:** 2018-10-26

**Authors:** Adriana S. A. Pereira, Murilo S. Amaral, Elton J. R. Vasconcelos, David S. Pires, Huma Asif, Lucas F. daSilva, David A. Morales-Vicente, Vitor C. Carneiro, Claudia B. Angeli, Giuseppe Palmisano, Marcelo R. Fantappie, Raymond J. Pierce, João C. Setubal, Sergio Verjovski-Almeida

**Affiliations:** 1 Laboratório de Parasitologia, Instituto Butantan, São Paulo, SP, Brasil; 2 Departamento de Bioquímica, Instituto de Química, Universidade de São Paulo, São Paulo, SP, Brasil; 3 Instituto de Bioquímica Médica Leopoldo de Meis, Universidade Federal do Rio de Janeiro, Rio de Janeiro, RJ, Brasil; 4 Instituto de Ciências Biomédicas, Departamento de Parasitologia, Laboratório de Glicoproteômica, Universidade de São Paulo, São Paulo, SP, Brasil; 5 Centre d'Infection et d'Immunité de Lille, CNRS UMR 8204, Inserm U1019, CHU Lille, Institut Pasteur de Lille, Université de Lille, Lille, France; University of Pennsylvania, UNITED STATES

## Abstract

**Background:**

The possibility of emergence of praziquantel-resistant *Schistosoma* parasites and the lack of other effective drugs demand the discovery of new schistosomicidal agents. In this context the study of compounds that target histone-modifying enzymes is extremely promising. Our aim was to investigate the effect of inhibition of EZH2, a histone methyltransferase that is involved in chromatin remodeling processes and gene expression control; we tested different developmental forms of *Schistosoma mansoni* using GKS343, a selective inhibitor of EZH2 in human cells.

**Methodology/Principal findings:**

Adult male and female worms and schistosomula were treated with different concentrations of GSK343 for up to two days *in vitro*. Western blotting showed a decrease in the H3K27me3 histone mark in all three developmental forms. Motility, mortality, pairing and egg laying were employed as schistosomicidal parameters for adult worms. Schistosomula viability was evaluated with propidium iodide staining and ATP quantification. Adult worms showed decreased motility when exposed to GSK343. Also, an approximate 40% reduction of egg laying by GSK343-treated females was observed when compared with controls (0.1% DMSO). Scanning electron microscopy showed the formation of bulges and bubbles throughout the dorsal region of GSK343-treated adult worms. In schistosomula the body was extremely contracted with the presence of numerous folds, and growth was markedly slowed. RNA-seq was applied to identify the metabolic pathways affected by GSK343 sublethal doses. GSK343-treated adult worms showed significantly altered expression of genes related to transmembrane transport, cellular homeostasis and egg development. In females, genes related to DNA replication and noncoding RNA metabolism processes were downregulated. Schistosomula showed altered expression of genes related to cell adhesion and membrane synthesis pathways.

**Conclusions/Significance:**

The results indicated that GSK343 presents *in vitro* activities against *S*. *mansoni*, and the characterization of EZH2 as a new potential molecular target establishes EZH2 inhibitors as part of a promising new group of compounds that could be used for the development of schistosomicidal agents.

## Author summary

Schistosomiasis is a chronic and debilitating disease caused by a trematode of the genus *Schistosoma*. The current strategy for the control of the disease involves treatment with praziquantel, the only available drug. The development of new drugs is therefore a top priority. Drugs that inhibit histone modifying enzymes have been used in cancer, altering gene expression, replication, repair and DNA recombination. *Schistosoma* parasites have some characteristics similar to malignant tumors, such as intense cell division and high levels of metabolic activity. Here we evaluate in *Schistosoma mansoni* the effect of GSK343, an inhibitor of the histone methyltransferase EZH2 that had been shown to arrest or reduce the growth of human cancer cells. We show that GSK343 causes damage to the parasite tegument and reduces egg laying in vitro, concomitant with a decrease in levels of H3K27me3, the histone mark put in place by EZH2. RNA-seq and proteomic analyses of treated parasites showed changes in the expression of hundreds of genes involved in important metabolic processes. In females, a marked decrease was observed in the expression of genes related to processes such as DNA replication and noncoding RNA metabolism. In conclusion, the histone methyltransferase EZH2 seems to be a promising novel drug target against schistosomiasis.

## Introduction

Schistosomiasis is a chronic and debilitating disease caused by trematodes of the genus *Schistosoma* and is one of the most prevalent and neglected diseases of tropical and subtropical regions, affecting more than 250 million people in 78 countries [1,2]. Despite efforts to control schistosomiasis in Brazil, it remains the South American country with the highest number of registered cases [3,4]. The severity of the disease and the organic deficit it produces make schistosomiasis the second most important neglected tropical disease in terms of death and morbidity, behind only malaria [4,5].

Current schistosomiasis treatment is based on the use of praziquantel (PZQ), which is effective against all *Schistosoma* species infecting humans [6]. Despite the advantages of PZQ as an anthelmintic drug of choice, which include tolerability, safety, efficacy, and low cost, it does not protect individuals against reinfection [7]. A major disadvantage of PZQ is its low efficacy against immature forms [8] and female worms [9]. In addition, the appearance of resistance of some strains of *Schistosoma* to the drug is a constant concern for the public health authorities [7,10,11]. Thus, there is a need for identifying new targets [12] and for planning, development and research of new compounds as potential schistosomicides [13].

*Schistosoma*, like other parasites, has some physiological characteristics similar to malignant tissues, such as intense and out-of-control cell division (for egg production), and a high level of metabolic activity similar to tumors [14]. Such metabolic similarities have generated interest in testing histone deacetylase inhibitor anti-cancer drugs such as valproic acid (VPA), suberoylanilide hydroxamic acid (SAHA) and Trichostatin A (TSA) as schistosomicidal compounds [14]. In particular, TSA has been shown to cause mortality of schistosomula and adults, an increase in apoptosis and increase in caspase 3/7 activity [14].

In our group, we have recently identified a set of genes differentially expressed in schistosomula exposed to TSA in culture [15], which are genes associated with DNA replication, recombination and repair, cell cycle and cell death. Interestingly, a network of genes with altered expression related to cell death was identified [15], among which the Embryonic Ectoderm Development (EED) gene was present; EED expression was strongly suppressed by TSA treatment [15]. EED encodes a regulatory protein that is required for the catalytic activity of the histone methyltransferase EZH2 enzyme, the subunit of polycomb repressor complex 2 (PRC2) that is responsible for trimethylation of histone H3 lysine 27 (H3K27me3) and inhibition of gene transcription [16]. Inhibition of EED expression by TSA led us to test the hypothesis that the decrease in the activity of PRC2 would be related to the death of the parasites [15]. We therefore targeted the *S*. *mansoni* PRC2 with GSK343 [15], a compound that is a known inhibitor of the human EZH2 enzyme [17,18] that was predicted to bind to SmEZH2 and act as a competitive inhibitor of its cofactor S-adenosyl methionine (SAM) [15]; prediction was done by homology modeling [15] of the SmEZH2 protein (Smp_078900) to the crystal structure of the human hEZH2 [19], followed by GSK343 docking to the SAM cofactor pocket of the SmEZH2 protein [15]. In that work, we showed that GSK343 has a synergistic effect along with TSA in promoting *S*. *mansoni in vitro* death [15].

In the present work we investigated in detail the effect of exposing *S*. *mansoni in vitro* to GSK343 alone. GSK343 was found to kill three different developmental forms of *S*. *mansoni* tested, namely schistosomula, adult males and females, and western blotting confirmed a decrease in the H3K27me3 histone mark in all three forms. Scanning electron microscopy and phenotypic parameters such as motility, viability, oviposition and mating were analyzed to document the changes upon GSK343 exposure that accompanied parasite death. Analysis of the effect of GSK343 on the large-scale gene expression of treated worms was performed with a high-throughput sequencing strategy (RNA-seq). In addition, the effect of GSK343 on the relative abundance of proteins was determined with quantitative mass spectrometry. Profiles of altered gene expression and of altered proteins abundance were identified.

## Methods

### Ethics statement

The experimental protocols were in accordance with the Ethical Principles in Animal Research adopted by the Brazilian College of Animal Experimentation (COBEA) and the protocol/experiments have been approved by the Ethics Committee for Animal Experimentation of Instituto Butantan (CEUAIB n° 1777050816).

### Maintenance of parasite life-cycle

The BH strain of *Schistosoma mansoni* (Belo Horizonte, Brazil) was maintained in the intermediate snail host *Biomphalaria glabrata* and as definitive host the golden hamster (*Mesocricetus auratus*). Female hamsters aged 3–4 weeks, freshly weaned, weighing 50–60 g, were housed in cages (30x20x13cm) containing a sterile bed of wood shavings. A standard diet (Nuvilab CR-1 Irradiada, Quimtia S/A, Paraná, Brazil) and water were made available *ad libitum*. The room temperature was kept at 22 ± 2°C and a 12:12 hour light–dark cycle was maintained. Hamsters were infected by exposure to a *S*. *mansoni* cercariae suspension containing approximately 200–250 cercariae using the ring technique [20]. After 49 days of infection, the *S*. *mansoni* adult worms were recovered by perfusion of the hepatic portal system [21]. Alternatively, after 21 days of infection juvenile worms [22,23] were collected by perfusion [21]. Cercariae were released from infected snails and mechanically transformed to obtain schistosomula *in vitro* [24].

### Treatment of schistosomula, juvenile and adult worms with GSK343

Schistosomula, juvenile worms and adult worms were treated with different final concentrations of GSK343 in culture medium specific to each stage as indicated in the Results (from a stock solution of 20 mM GSK343 in DMSO), and with the equivalent amount of DMSO in the control assays. Newly transformed schistosomula (NTS) were maintained for 16 h in M169 (Vitrocell) medium supplemented with 2% fetal bovine serum (FBS) (Vitrocell), 1 μM serotonin, 0.5 μM hypoxanthine,1 μM hydrocortisone, 0.2 μM triiodothyronine, penicillin/streptomycin, amphotericin, gentamicin (Vitrocell) at 37°C and 5% CO_2_ [25]. Only after 16h incubation with the culture medium was the GSK343 treatment initiated. Juvenile worms and paired adult worms were maintained in RPMI medium (Gibco) supplemented with 10% fetal bovine serum (FBS) (Vitrocell), penicillin/streptomycin, amphotericin, gentamicin (Vitrocell) at 37°C and 5% CO_2_.

### Motility assay

An inverted microscope was used to evaluate the general condition of adult worms, including motility and mortality rate. Parasites were observed after 3, 6, 12, 24 and 48 h of treatment with 20 μM GSK343 or vehicle dimethyl sulfoxide (DMSO 0.1%). The motility and survival of worms were assessed according to the criteria scored in a viability scale of 0–3 [26]. The scoring system was as follows: 3, complete body movement; 1.5, partial body movement or immobile but alive; and 0, dead. Treatment was considered lethal whenever no worm movement was detected when observed for more than 2 min. In addition, pairing status and oviposition were recorded.

### Viability assay

The viability of schistosomula and *S*. *mansoni* adult worms after treatment was determined by a cytotoxicity assay based on the CellTiter-Glo Luminescent Cell Viability Assay (G7570, Promega, Madison, Wisconsin, EUA) [27]. The assay determines the amount of ATP present in freshly lysed adults or in intact schistosomula; the assay signals the presence of metabolically active cells.

In addition, viability of schistosomula was evaluated by the presence or absence of dead parasites, through staining with propidium iodide (PI) [28] and fluorescein diacetate (FDA) [29], as follows. Schistosomula were equally distributed in 96-well microtiter plates, incubated with the concentration of GSK343 indicated in the figure or the corresponding DMSO vehicle (control), and 2 μg/mL propidium iodide (PI) (Sigma-Aldrich) plus 0.5 μg/mL fluorescein diacetate (FDA) (Life Technologies) were added at the time points indicated in the legend to the figure. The parasites were immediately observed with light microscopy at 10 x magnification using a Nikon Eclipse fluorescent inverted microscope. Under light microscopy, viable parasites were scored by preserved mobility and lack of opacity. Under fluorescence microscopy, schistosomula death was scored by a red fluorescence signal (572 nm emission microscope filter); in living schistosomula cells FDA is converted into charged fluorescein by parasite esterase activity, staining the schistosomula with a green fluorescence signal (492 nm emission microscope filter) [28,29]. For each time point a new set of wells was used, because the staining procedure was lethal to the parasites. The number of biological replicates that were assayed, as well as the number of parasites that were counted per replicate, is stated in the legends to the figures.

### Caspase 3/7 activity

Caspase 3/7 activity was measured using the Caspase-Glo 3/7 assay kit (Promega) following manufacturer’s instructions. Briefly, 2,000 schistosomula per well were cultivated in a 24 well plate with complete medium (M169 medium supplemented as described above) plus 20 μM GSK343 or vehicle (DMSO 0.1%). At 48 h incubation the medium was replaced, the reporter lysis buffer was added, followed by 3 h incubation with the Caspase-Glo reagent, in the dark with agitation. A negative control was carried out without the Caspase-Glo reagent. Luminescence was measured in a white-walled 96-well plate in a Wallac Victor2 1420 multilabel counter (PerkinElmer).

### TUNEL assay

Detection of DNA strand breaks in GSK343-treated schistosomula was performed using the In situ Cell Death Detection kit (Roche) [30]. Briefly, 2,000 schistosomula per well were cultivated in a 24-well plate with complete medium (M169 medium supplemented as described above) plus 20 μM GSK343 or vehicle (DMSO). At 72 h incubation, the schistosomula were fixed for 60 min, made permeable prior to labeling as recommended by the manufacturer and mounted in a glass slide using Prolong with DAPI (Invitrogen, USA) for nuclear visualization. Images were taken on a Zeiss Axio Observer Z1 inverted microscope equipped with a 40× objective lens and an AxioCam MRm camera, in the ApoTome mode.

### Scanning electron microscopy (SEM)

Adult and juvenile worms collected by perfusion were immediately transferred to supplemented RPMI medium. The parasites were distributed in 24-well plates (adults: one paired worm couple per well; juveniles: five worms per well) with the same medium. The schistosomula were distributed in 6-well plates (2,000 per well) containing the supplemented M169 medium. The worms were kept in culture (oven at 37°C and 5% CO_2_) for 2 h for adaptation, and then GSK343 was added.

Ultrastructural analysis was performed with scanning electron microscopy. Adult worms, juvenile worms and schistosomula incubated in 20 μM GSK343 or DMSO vehicle for 24 h and 48 h were fixed with modified Karnovsky reagent (1% paraformaldehyde, 2.5% glutaraldehyde, 1 mM calcium chloride in 1 M sodium cacodylate buffer, pH 7.4) and after the fixing stage the material was washed with sodium cacodylate buffer (0.1 mol / L, pH 7.2) and post-fixed with 1% osmium tetroxide (w / v) for 1 h. The same protocol was used for SEM of eggs released from the couples incubated with GSK343 or the vehicle.

The samples were dehydrated with increasing concentrations of ethanol and then dried with liquid CO_2_ in a critical-point dryer machine (model Leica EM CPD030, Leica Microsystems, Illinois, USA). Treated specimens were mounted on aluminum microscopy stubs and coated with gold particles using a ion-sputtering apparatus (model Leica EM SCD050, Leica Microsystems, Illinois, USA) [31]. Specimens were then observed and photographed using an electron microscope (FEI QUANTA 250, Thermo Fisher Scientific, Oregon, USA).

### Western blotting

In the preparation of histone acid extracts of adult parasites, fifty pairs of worms (either treated or control) were soft lysed with 500 μL lysis buffer (PBS containing 0.5% Triton X-10, 0.02% NaN_3_ and Mini Protease Inhibitor Cocktail-Complete from Roche) in a glass Potter homogenizer. The samples were centrifuged (10 min, 2000 g at 4°C) and pellets containing the nuclear material were washed once in 200 μL lysis buffer then centrifuged again [14]. The pellet containing the nuclear fraction with the histones was resuspended in 400 μL 0.25 M HCl with protease inhibitor and the solution was incubated overnight at 4°C in order to precipitate acid proteins [32]. The samples were centrifuged (30 min, 16000 g at 4°C) and the histone proteins contained in the supernatant were concentrated with trichloroacetic acid 33% [33]. The final pellet with histones was taken up in MilliQ water with protease inhibitors.

For schistosomula nuclear histones extraction, treated or control samples containing approximately 10,000 schistosomula each were used. Each sample was sonicated on ice in an Eppendorf tube for 10 min with an Active Motif sonicator model Q120AM (5 s ON, 5 s OFF, 20% amplitude) in lysis buffer (PBS containing 0.5% Triton X-100, 2 mM PMSF, 0.02% NaN_3_ and Mini Protease Inhibitor Cocktail—Complete from Roche). After the sonication, total lysis of the schistosomula was checked under an optical microscope, the samples were centrifuged 20 min at 13,000 g, 4°C, and the supernatant containing the nuclear protein extract was stored in a clean tube at -80°C.

The nuclear protein concentration of extracts of adult worms or schistosomula were determined with the Micro BCA Protein Assay kit (Pierce Biotechnology). From each sample, 10 μg of extracts was loaded on 15% SDS-Polyacrylamide gels, and after electrophoretic protein separation, the proteins were electro-transferred (TE 77 Amersham Biosciences) from the gel to a nitrocellulose membrane (Amersham) for 1.5 h at 100 mA. Briefly, membranes were blocked with Tris-buffered saline (TBS) containing 0.1% Tween 20 and 2% bovine serum albumin (TBST/2% BSA), and then probed overnight with specific antibodies in TBS/2% BSA. Membranes were washed with TBST and incubated for 1 h in TBST/2% BSA with secondary antibody conjugated with IRDye (IRDye 800CW goat anti-rabbit or IRDye 700CW goat anti-mouse from Licor Biosciences). After washing the membranes in TBST, the bands were visualized and images were recorded with the Odyssey Infrared Imaging System (Licor Biosciences), and quantified with Image J [34]. Acetylation and methylation of histones was measured with specific monoclonal antibodies to the following lysine modifications: Histone H3 acetyl K9 antibody C5B11 (Cell Signaling) (1:1000), Histone H3 acetyl K27 antibody ab45173 (Abcam) (1:500), Histone H3 tri-methyl K4 antibody 04–745 (Millipore) (1:2000), Histone H3 tri-methyl K27 antibody ab6002 (Abcam) (1:1000), Histone H3 mono-methyl K27 antibody 61015 (Active Motif) (1:250). For normalization of the signals across the samples anti-Histone H3 antibody ab24834 (Abcam) (1:1000) was used.

### RNA extraction for deep sequencing (RNA-Seq)

Adult worms or schistosomula were maintained in the stage-specific culture media described above (at 37°C and 5% CO_2_) and were treated for 48 h with either 20 μM GSK343 or the DMSO (0.1%) vehicle.

Total RNA extraction from parasites was performed using the RNeasy Mini Kit (QIAGEN) for adults (in three biological replicates) and the RNeasy Micro Kit (QIAGEN) for schistosomula (four biological replicates), following the instructions provided by the manufacturer, including the optional DNase treatment during extraction of total RNA to ensure the removal of eventual contaminating genomic DNA present in RNA.

RNA purity was evaluated with NanoDrop (ND-100 Spectrophotometer, Thermo Scientific) and it was adequate for RNA-seq library construction (ratio A260 / A280 = 2.2, A260 / A230 = 2.0). RNA integrity was assessed by electrophoresis with the 2100 Bioanalyzer (Agilent). The Agilent RNA 6000 Nano or Pico Assay protocols were followed, depending on the amount of RNA in the samples. Only the band corresponding to 18S ribosomal RNA and not the 28S band was present, as expected [35], plus a smear above 500 nt, which indicated no RNA degradation. The RNA concentration was measured with Qubit 2.0 fluorimeter (Invitrogen by Life Technologies), and the total RNA obtained was in sufficient quantity (> 1 μg per sample of adult worms) for library construction with the KAPA Stranded mRNA-Seq Kit (KK8421) for adult worm samples, and with the SMART-Seq v4 Ultra Low Input RNA Kit (634891) for schistosomula samples (> 10 ng per schistosomula sample). Both kits have steps where polyA+ RNA is purified prior to cDNA generation. Library construction and RNA-Seq were performed in biological triplicates for adult worms and in quadruplicates for schistosomula at the Duke Center for Genomic and Computational Biology facility (Duke University Durham, NC, USA). Sequencing was on the Illumina HiSeq 2500 platform.

### RNA-seq dataset collection and preprocessing

The Illumina sequencing data (raw reads) were downloaded from the FTP server at the Duke University facility and verified for files integrity with MD5 checksum. A total of 22 to 32 million reads was obtained per each replicate sample of adult worms and 11 to 13 million reads per each replicate sample of schistosomula. Quality check of reads was with FastQC (www.bioinformatics.babraham.ac.uk/projects/fastqc/), and Trimmomatic [36] was used to trim Illumina adapters and to filter out reads with low quality values using the following parameters: MAXINFO with parameters 50:0.05, HEADCROP of 28 nucleotides, a LEADING and TRAILING value of 5, and minimum read length of 30 nucleotides.

### Differential expression analyses from RNA-seq data

Mapping of reads to the genome and gene differential expression analysis were performed with three different pipelines as described below. The different tools handle the problem of mapping reads to alternatively spliced genes in different ways; the intersection of results of differentially expressed genes (DEGs) from the three analyses was used as the core, most significant DEGs. Version 5.2 of the *S*. *mansoni* genome [37] was used for all analyses.

The first pipeline used Tophat2 [38] that is part of the Tuxedo Tools, along with a custom reference transcriptome gff file, which was based on the Sanger transcriptome [37] and includes corrections for inconsistencies in some coordinates of start and end of UTRs; this custom reference transcriptome gff file is available at http://verjolab.usp.br. Read counting was done with cuffquant using the Tophat2 BAM files as input, and a metagenes transcriptome [39] as reference; this metagenes transcriptome reference gtf file is available at http://verjolab.usp.br. Statistical significance analysis of differential expression was obtained by using cuffdiff [40] with cuffquant counting as input and the default parameters, which imply the pooled dispersion method and the geometric library normalization method. A q-value ≤ 0.05 was used as significance threshold.

The metagenes transcriptome reference takes into account the evidence from public RNA-seq data either that two or more genomic neighbor Smp_nnnnnn predicted genes do encode fragments of the same protein and should be merged, or that in a given genomic locus a novel transcript was assembled from the public RNA-seq data [39]. In the two cases the corresponding transcript was named “SmVG_xxxxxx”, where xxxxxx stands for the XLOC_xxxxxx identifier number generated for that locus during the assembly [39]. In the case that one or more Smp genes comprise the SmVG_xxxxxx metagene, it was further annotated as SmVG_xxxxxx:Smp_nnnnnn to indicate the corresponding Smp_nnnnnn genes that comprise that SmVG_xxxxxx; the expression value of that metagene was assigned to all SmVG_xxxxxx:Smp_nnnnnn genes belonging to the same SmVG_xxxxxx metagene. Sequence and genomic coordinates information for all SmVG_xxxxxx genes is available at the metagenes transcriptome reference gtf file mentioned above.

The second pipeline employed Kallisto [41], which uses a pseudo-alignment approach to map the reads against the genome. The metagenes transcriptome was again used as reference. Sleuth [41] statistical package was used to find statistically significant DEGs with a q-value ≤ 0.05.

Finally, the third pipeline employed Sailfish [42], which uses a quasi-mapping approach to map the reads against the genome. EdgeR [43] was used for the statistical analyses and the exact test proposed by Robinson and Smyth [44] was selected. A q-value ≤ 0.05 was used as significance threshold.

As mentioned, we retained for further processing only the consensus DEGs found by all three procedures described above. Detailed expression values for all replicates and differential expression statistical significance for these consensus genes are available at http://verjolab.usp.br. Simplified lists with gene IDs, q-values, GO terms and product description are given in the tables indicated in the Results section below.

### Gene Ontology enrichment analyses

Gene enrichment analysis was performed with BINGO [45] using the *S*. *mansoni* annotated Gene Ontology (GO) terms (www.geneontology.org) and the list of DEGs for each tested condition. An enrichment cutoff value of corrected p-value ≤ 0.05 was used as significance threshold.

### RT-qPCR validation of DEGs

For quantitative RT-PCR, complementary DNAs were obtained by reverse transcription (RT) of 100 ng schistosomula or adult worms total RNA using SuperScript III Reverse Transcriptase (Invitrogen) and random hexamer primers in a 20 μL volume, according to the manufacturer’s recommendations. The resulting cDNA was diluted 8-fold in water and qPCR amplification was done with 2.5 μL of diluted cDNA in a total volume of 10 μL using SYBR Green Master Mix (Life Technologies) and specific primer pairs (S1 Table) designed for *S*. *mansoni* genes by Primer3 online software. The Light cycle 480 II (Roche) qPCR was used. The results were analyzed by comparative Ct method and the statistical significance was calculated with the t-test.

To find adequate normalizer genes for qPCR, we looked for evidence of genes with non-detectable changes in expression upon GSK343 treatment in the RNA-Seq data. For this, genes with normalized TPM values > 1 in the control samples, as computed both by Sailfish and by Kallisto (see above) were selected. Further filtering retained the genes that were not in the list of DEGs, and had an average TPM ratio treated/control in both Sailfish and Kallisto analyses within the interval 0.9–1.1 (maximum allowed difference between control and treated expression is 10%); additionally, the selected genes in the adult females and males analysis were pooled and ranked by their coefficient of variation, from smaller to larger, and the top 5 genes were selected for testing by qPCR; for schistosomula, the top 4 genes in a similar analysis were selected for testing by qPCR. The final set of normalizers was confirmed by qPCR as having their expression ratio within the interval 0.9–1.1. For adult worms, the normalizers were granulin (Smp_170550), ATP synthase subunit beta (Smp_029390) and serine threonine protein phosphatase 2A (Smp_166290). For schistosomula, the normalizers were mitochondrial ATP synthase B subunit (Smp_154530) and casein (Smp_025010). In all cases, the average expression of normalizers was used for calculating the expression of the genes of interest.

### Protein extraction and digestion for mass spectrometry

Schistosome parasites were lysed in lysis buffer containing urea (8 M), Mini Protease Inhibitor Cocktail-Complete from Roche and 50 mM ammonium bicarbonate (Ambic). Extracted proteins were quantified using Micro BCA Protein Assay kit (Pierce Biotechnology), reduced with dithiothreitol (10 mM) for 30 min at room temperature and subsequently alkylated with iodoacetamide for 30 min in the dark. Following incubation, the samples were diluted in 20 mM Ambic, pH 7.5, and digested with trypsin (Promega V5111) 1:50 (w/w) overnight at room temperature. Samples were acidified with trifluoroacetic acid (TFA) (1% final concentration) and centrifuged at 14,000 *g* for 10 min to stop trypsin digestion. Supernatant was collected and dried prior to desalting. Three biological replicates for treated and untreated conditions were processed and analyzed.

### Peptide desalting for mass spectrometry

Samples were resuspended in 0.1% TFA and desalted using homemade micro-columns comprised of a C18 plug taken from a C18 disk (Sigma-Aldrich) with the constricted end of a P200 tip inserted into the disk. The acidified samples were loaded onto the micro-column by applying a gentle air pressure with the syringe and washed three times with 0.1% TFA. Peptides were eluted with 50% acetonitrile (ACN)/ 0.1% TFA, followed by 70% ACN/ 0.1% TFA.

### Mass spectrometric analyses

Peptide samples were dried in the Speed-Vac, resuspended in 0.1% formic acid (FA) and analyzed using an EASY-nLC liquid chromatography system (Thermo Scientific) coupled to LTQ-Orbitrap Velos mass spectrometer (Thermo Scientific). The peptides were loaded on a Reprosil-Pur C18-AQ (3 μm) column and separated in an organic solvent gradient from 100% phase A (0.1% FA) to 30% phase B (0.1% FA, 95% ACN) during 80 min for a total gradient of 105 min at a constant flow rate of 300 nL/min. The LTQ-Orbitrap Velos was operated in positive ion mode with data-dependent acquisition. The full scan was acquired in the Orbitrap with an automatic gain control (AGC) target value of 10e6 ions and a maximum fill time of 500 ms. Peptides were fragmented by collision-induced dissociation. Ions selected for tandem mass spectrometry (MS/MS) were dynamically excluded for a duration of 30 s. Each MS scan was acquired at a resolution of 60,000 FWHM followed by 20 MS/MS scans of the most intense ions. All raw data were accessed with the Xcalibur software (Thermo Scientific).

### Protein database searches and analyses

Raw data were processed using MaxQuant software version 1.5.2.8 and the embedded database search engine Andromeda [46]. A custom reference Proteins Database was used (http://verjolab.usp.br); this fasta file contains the >Smp_nnnnnn protein sequences [37] from Ensembl (http://metazoa.ensembl.org/Schistosoma_mansoni/Info/Index) plus the 212 novel protein sequences (>cnnnn-gn-in) described by Anderson *et al*. [15] and the micro-exon genes (>MEGnnnnnn and >GUnnnnnn sequences) described by DeMarco *et al*. [47]. The MS/MS spectra were searched against this custom reference database with the addition of common contaminants, with an MS accuracy of 4.5 ppm and 0.5 Da for MS/MS. Cysteine carbamidomethylation (57.021 Da) was set as the fixed modification, and two missed cleavages for trypsin. Methionine oxidation (15.994 Da) and protein N-terminal acetylation (42.010 Da) were set as variable modifications. Proteins and peptides were accepted at FDR less than 1%. All raw data have been submitted to the PRIDE archive [48,49].

Label-free quantitation (LFQ) was performed using the MaxQuant software with the “match between runs” feature activated. Protein LFQ intensity of each of the three biological replicates of treated and untreated samples were used for further statistical analyses. Proteins with valid values in at least two replicates of each biological condition were kept in the analyses. Exclusive proteins were identified in at least two biological replicates of one condition and in none of the other. Significantly regulated proteins were determined using the Welch’s t-test using the Perseus v.1.5.1.6 software [50], and a p ≤ 0.05 was used as significance threshold.

## Results

### Phenotypic effects of GSK343 on *S*. *mansoni* adult worms and eggs

**Fig 1:**
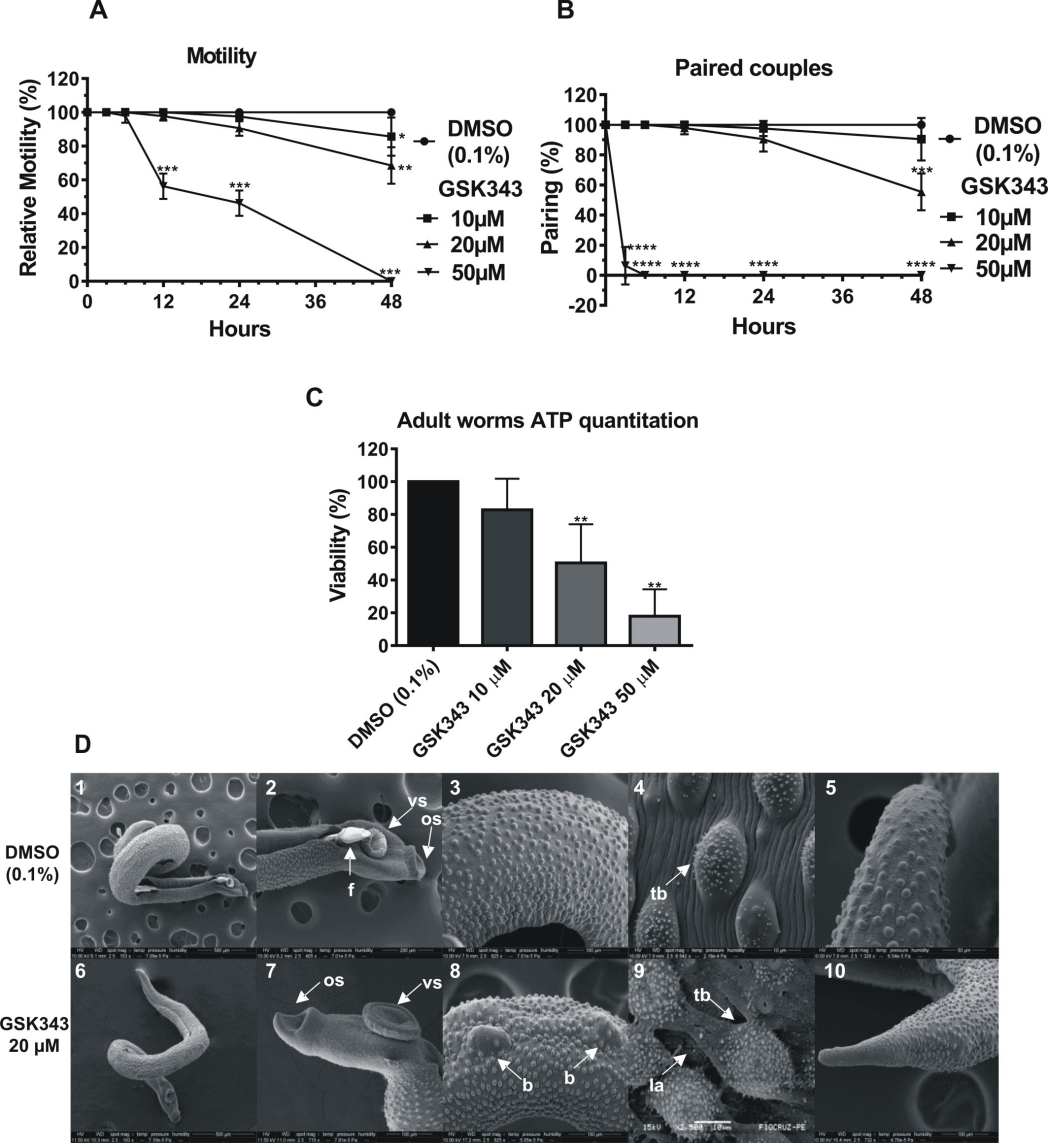
*In vitro* effects of GSK343 on the motility, pairing, viability and ultrastructure of *S*. *mansoni* adult worms. **(A)** Percentage of relative motility of adult worms treated with different concentrations of GSK343 and controls (0.1% DMSO) at different times of exposure from 3 to 48 h. Mean ± SD of three experiments, each with 10 worm pairs. **(B)** Monitoring the pairing of controls and treated couples with different concentrations of GSK343 at different times of exposure from 3 to 48 h. Mean ± SD of three experiments, each with 10 worm pairs. **(C)** Viability was estimated by the total amount of ATP available in the parasites, using a luminescent assay. Pairs of adult worms were treated for 48 h with GSK343 at the different concentrations indicated or with vehicle (0.1% DMSO). The viability was expressed as % the luminescence values relative to the control (DMSO). Mean ± SD from three replicate experiments, each with 10 worm pairs. For panels **(A)**, **(B)** and **(C)**: *p < 0.05, **p < 0.01, ***p < 0.001, ****p < 0.0001 compared with control. **(D)** Scanning electron microscopy of worms exposed for 48 h to 20 μM GSK343 (note that the size bar is shown within the black thin line below each image); **panels 1 and 2**: Male and female control (Bar = 500 μm; 200 μm) presenting the couple fully paired; **panels 3 and 4**: Medial dorsal region of the male presenting tegument without structural alterations (Bar = 100 μm; 10 μm); **panel 5**: Male posterior region without morphological alterations (Bar = 50 μm). **panels 6 and 7**: Adult worm treated with GSK343 and its anterior region (Bar = 500 μm; 100 μm); **panels 8 and 9**: Enlarged view of dorsal region of adult worm treated with GSK343 showing lesions of tegument around the tubercles; **panel 10**: Posterior region of male adult worm treated with GSK343 (Bar = 100 μm). *f*: *female; os*: *oral sucker; vs*: *ventral sucker; b*, *blebs; tb*: *tubercles; la*: *lesion area*.

The *S*. *mansoni* adult worms exhibited decreased motility when exposed to GSK343, and this was dependent upon concentration and incubation time (Fig 1A). After 48 h the adult worms lost approximately 40% of their motility at the sublethal dose of 20 μM GSK343 (Fig 1A), there was impairment of the peristaltic movement and the ability of their suckers to adhere to the bottom of the culture plates. When exposed to 50 μM GSK343, the motility of the worms was altered after 12 h of incubation (Fig 1A). At 24h the motility was further decreased to 50%, and some dead worms were already observed. At 48 h with 50 μM GSK343, all worms were classified as dead (Fig 1A). Pairing of the couples was reduced to approximately 50% in relation to the control with 20 μM GSK343 after 48 h of exposure (Fig 1B). With 50 μM GSK343 pairing was drastically reduced to nearly zero already at 3 h of exposure (Fig 1B). Viability measured by ATP quantitation in adult worms after 48 h exposure to the compound (Fig 1C) was reduced to 50% in parasites treated with 20 μM GSK343 compared with controls (DMSO), and it was further reduced to less than 20% with 50 μM GSK343.

Throughout the observation intervals (3, 6, 12, 24 and 48 h), *S*. *mansoni* adult worms exposed to vehicle (DMSO 0.1%) exhibited normal peristaltic movements and characteristic waves along the body axis, suckers in constant movement, showing occasional adherence to the bottom of the culture plate through the ventral sucker; Pairing was maintained in the controls (Fig 1A and 1B).

With scanning electron microscopy (SEM), it was possible to detect the phenotypic differences between control and treated male adult worms. The tegument of control adult worms showed no morphological and structural changes (Fig 1D, panels 1 to 5). With 20 μM GSK343, after 48 h exposure, the presence of blebs in the dorsal region was observed, causing the tegument to appear distended (Fig 1D, panel 8) in some parts, possibly due to ballooning of the subtegumental region, and this was a constant characteristic present in all adult worm males analyzed with SEM. Areas of lesion near the tubercles were sometimes visualized, with fissures in the tegument surface and the presence of deep chasms between the dorsal tubercles (Fig 1D, panel 9). At the anterior and posterior regions of the parasite, no alterations were detected (Fig 1D, panels 6, 7 and 10).

**Fig 2:**
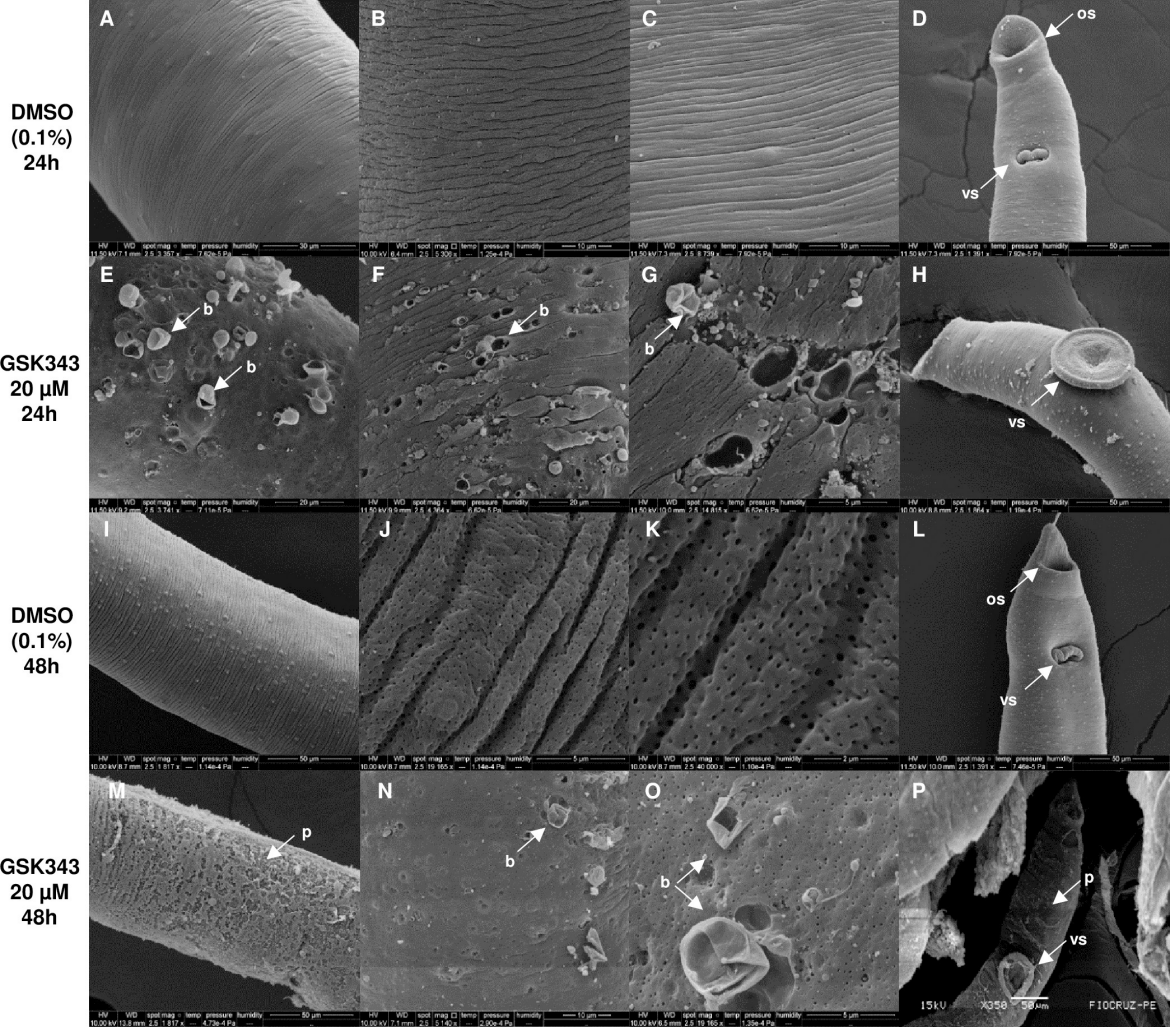
Damaged dorsal surface of *S*. *mansoni* adult female worms exposed to 20 μM GSK343 for 24 and 48 h. Scanning electron microscopy of parasites; note that the size bar is shown within the black thin line below each image. **(A, B and C)** Images of dorsal region of female control worms, incubated with DMSO (0.1%) for 24 h, without structural alterations (Bar = 30 μm; 10 μm and 10 μm).; **(D)** Anterior region of female control worm incubated for 24 h with vehicle (Bar = 50 μm); **(E, F and G)** Middle dorsal region of females incubated with GSK343 for 24 h presenting apparent surface damage of the membrane with the presence of several blisters in the tegument (Bar = 20 μm; 20 μm and 5 μm); **(H)** Anterior region of female worm treated with GSK343 for 24 h without structural alterations (Bar = 50 μm); **(I, J and K)** Dorsal region of female control worms, incubated with DMSO (0.1%) for 48 h presenting the entire tegument without changes (Bar = 50 μm; 5 μm and 2 μm); **(L)** Anterior region of female control worm incubated for 48 h with 0.1% DMSO showing no alterations. (Bar = 50 μm); **(M, N and O)** Middle dorsal region of females incubated with GSK343 for 48h showing peeling of the tegument and presence of blisters (Bar = 50 μm; 10 μm and 5 μm); **(P)** Anterior region of female worm treated with GSK343 for 48 h presenting peeling of the tegument and the ventral sucker (Bar = 50 μm). *os*: *oral sucker; vs*: *ventral sucker; b*: *blisters; p*: *peeling*.

**Fig 3:**
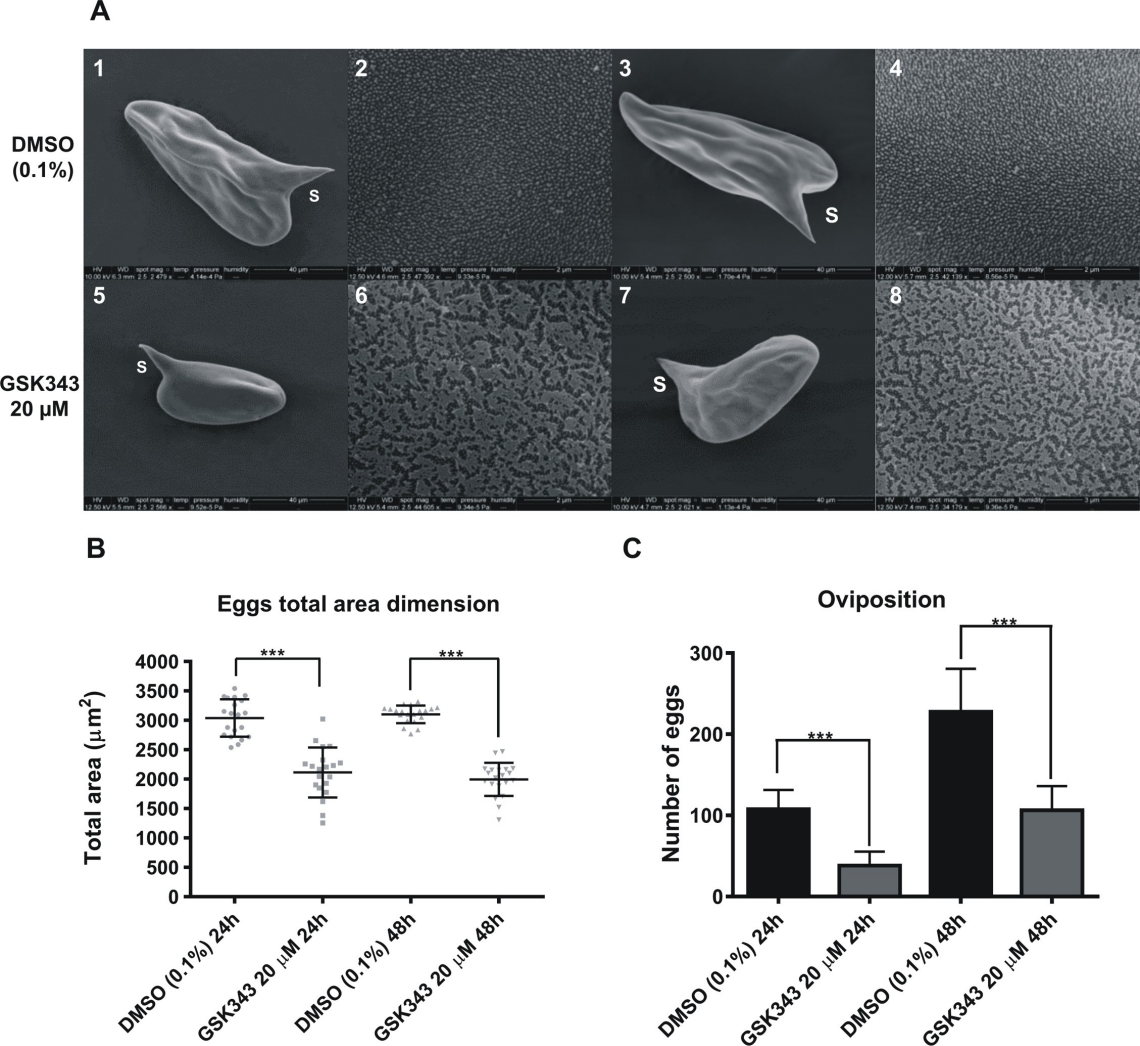
Decrease in egg size and oviposition in *S*. *mansoni* females exposed to 20 μM GSK343 for 24 or 48 h. Scanning electron microscopy of eggs; note that the size bar is shown within the black thin line below each image. **(A)** Micrographs of eggs from control females not exposed to the compound (0.1% DMSO); **panels 1 and 3:** Oval egg with normal lateral spine (*s*) at 24 and 48 h (Bar = 40 μm); **panels 2 and 4:** the surface of the eggs shows normal micro spicules at 24 and 48 h incubation (Bar = 2 μm). Micrographs of eggs from females exposed to GSK343 (20 μM); **panels 5 and 7:** Reduced egg size with lateral spine (*s*) at 24 and 48 h of treatment (Bar = 40 μm); **panels 6 and 8:** the surface of the eggs show fissures at 24 and 48 h (Bar = 2 μm; 3 μm). (**B and C)** Total area dimension of *S*. *mansoni* eggs and number of eggs released by females incubated with GSK343 (20 μM) or vehicle (0.1% DMSO), respectively. *** p < 0.001.

In females treated with 20 μM GSK343 for 24 or 48 h of incubation, severe damage to the tegument is visualized (Fig 2) exhibiting extensive sloughing that exposes subtegumental tissues. The presence of peeling (Fig 2M) and blisters can be seen throughout the extension of the dorsal region of the female (Fig 2E, 2F and 2N). In Fig 2G and 2O, the larger magnification permits visualization of the rupture of blisters where possibly there was extravasation of cytoplasm. The anterior region of the females presented damage to the tegument only after 48 h of incubation with the compound (Fig 2P). In addition, after 24 or 48 h of incubation with 20 μM GSK343 there was a decrease of about 40 to 60% in the number of eggs laid by these females in relation to the control (Fig 3C). It is worth pointing out that the percentage reduction in egg laying remains fairly constant from 24 to 48 h treatment, in contrast to the observed increase in lesions on the worms.

SEM of eggs released by females incubated with GSK343 was performed (Fig 3A, panels 5 to 8). These eggs were phenotypically different from control eggs (Fig 3A, panels 1 to 4). They showed fissures in the shell (Fig 3A, panels 6 and 8) and a 30–40% reduction in area (Fig 3B).

### Phenotypic effects of GSK343 on *S*. *mansoni* juvenile worms

**Fig 4:**
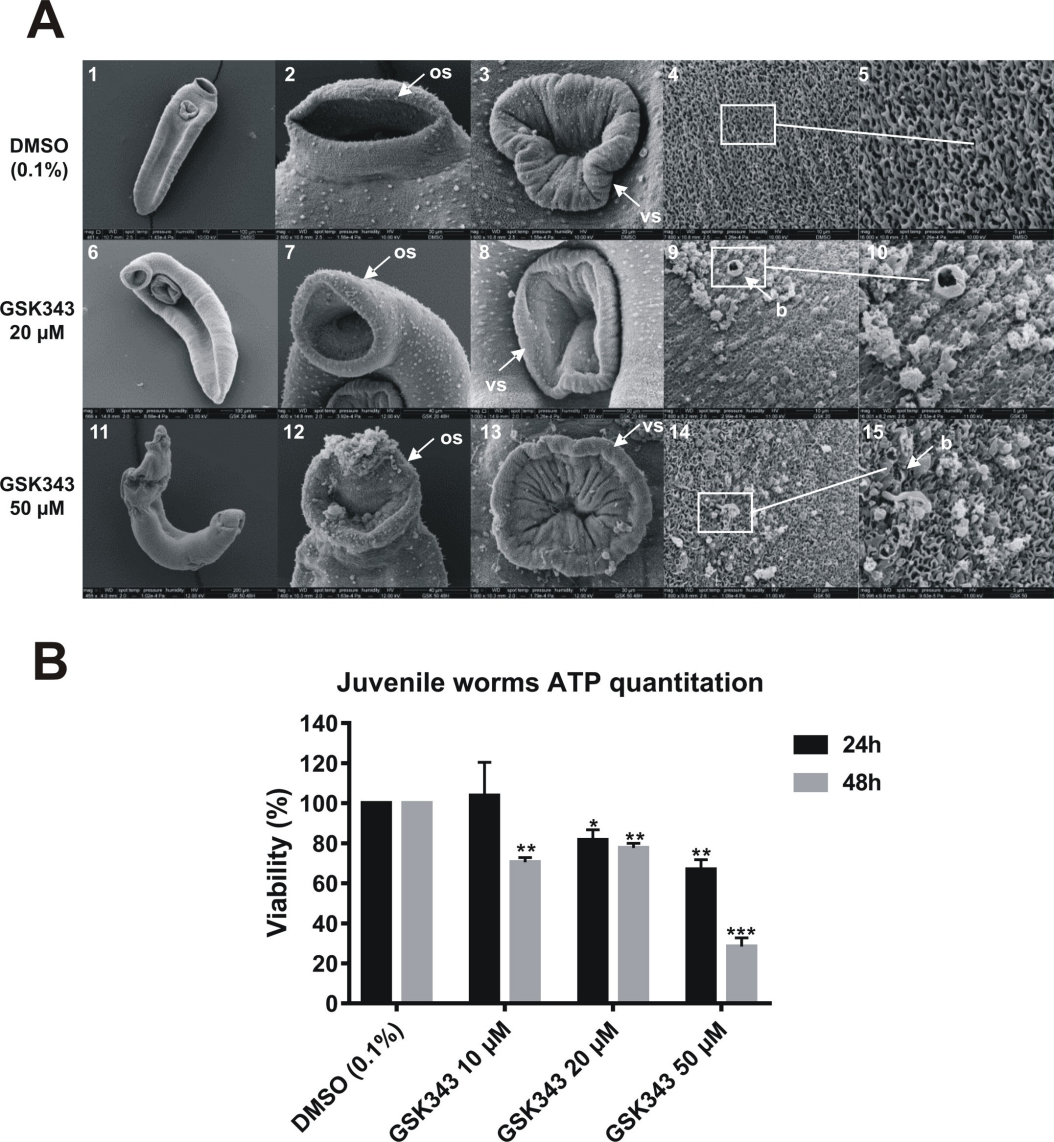
Predominant ultrastructural changes on the dorsal surface of *S*. *mansoni* juvenile worms exposed for 48 h to different concentrations of GSK343. **(A)** Scanning electron microscopy of juvenile parasites; note that the size bar is shown within the black thin line below each image. **(panel 1)** Low magnification image of control juvenile worm (0.1% DMSO) (Bar = 100 μm); **(panels 2 and 3)** Oral and ventral sucker of control juvenile worm presenting tegument without structural alterations (Bar = 30 μm and 20 μm); **(panels 4 and 5)** Medial dorsal region of control juvenile worms showing intact tegument (Bar = 20 μm and 5 μm); **(panel 6)** Low magnification image of juvenile worm treated with 20 μM GSK343 (Bar = 100 μm); **(panels 7 and 8)** Non-detectable changes to oral and ventral sucker of juvenile worm treated with 20 μM GSK343 (Bar = 40 μm and 30 μm); **(panels 9 and 10)** Medial dorsal region of juvenile worms showing enlarged view showing blisters and focal lesions on the tegument (Bar = 10 μm and 5 μm); **(panel 11)** Low magnification image of juvenile worm treated with 50 μM GSK343 shows altered morphology (Bar = 200 μm); **(panels 12 and 13)** Oral and ventral sucker of juvenile worm treated with 50 μM GSK343 presenting areas of cracking (Bar = 40 μm and 30 μm); **(panels 14 and 15)** Medial dorsal region of juvenile worms treated with 50 μM GSK343 showing erosion of tegument (Bar = 10 μm and 5 μm). *os*: *oral sucker; vs*: *ventral sucker; b*: *blisters*. **(B)** Viability of juvenile worms was estimated by the total amount of ATP available in the parasites, using a luminescent assay. Juvenile worms were treated for 24 h (black bars) and 48 h (gray bars) with GSK343 at the different concentrations indicated or with vehicle (0.1% DMSO). The viability was expressed as % of the luminescence values relative to the control (DMSO). Mean ± SD from three replicate experiments, each with 10 juvenile worms. *p < 0.05, ** p< 0.01, *** p< 0.001.

*S*. *mansoni* juvenile forms were treated with GSK343 and observed by SEM (Fig 4). In control (untreated) parasites the tegument appeared intact, the anterior region presented no structural alterations (Fig 4, panels 1 to 3) and at high magnification, the surface had a spongy appearance without morphological changes (Fig 4, panels 4 and 5).

In juvenile worms treated with GSK343 (20 and 50 μM) the dorsal surface ridges were severally damaged, they presented blisters in much of the dorsal tegument, with sloughing and erosion (Fig 4, panels 9, 10, 14 and 15). Anterior region damage was seen only in worms treated with 50 μM GSK343, with the oral sucker partially destroyed (Fig 4, panels 11 to 13). The viability of juvenile worms was measured by the quantitation of total ATP in the parasites. At 24 h an approximate 20 to 30% decrease in viability of parasites was observed when treated with 20 μM and 50 μM GSK343 (Fig 4B, black bars). At 48 h exposure of juvenile parasites to GSK343 an approximate 20 to 30% decrease in viability was already observed at 10 and 20 μM GSK343 (Fig 4B, gray bars). At 50 μM GSK343 a 70% decrease in viability of juvenile worms was observed (Fig 4B, gray bar).

### Phenotypic effects of GSK343 on *S*. *mansoni* schistosomula

**Fig 5:**
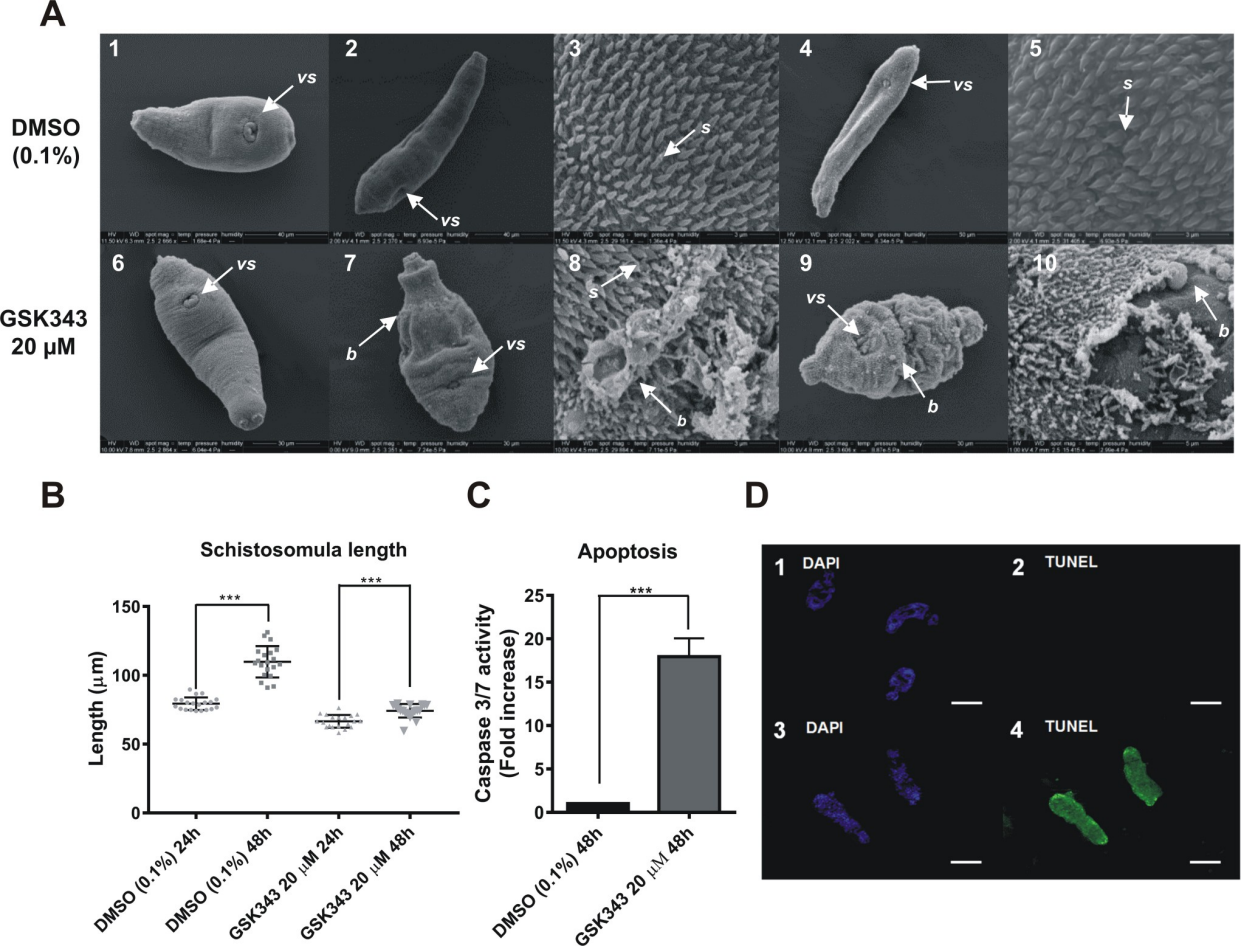
Morphological changes and increased apoptosis in schistosomula exposed to 20 μM GSK343. **(A)** Scanning electron microscopy of schistosomula; note that the size bar is shown within the black thin line below each image. **(A, panels 1 and 2)** Control schistosomula micrographs at the same magnification (Bar = 40 μm) showing the ventral sucker (*vs*) of typical schistosomula maintained for 16 h in culture medium (panel 1) or further incubated for 24 h in culture medium plus vehicle (0.1% DMSO) (panel 2); the lateral and ventral aspects are normal, and the schistosomula underwent normal development and lengthening after 24 h incubation in vehicle-containing medium (compare panels 2 and 1). **(A, panel 3)** Higher magnification of control schistosomulum from panel 2 showing that the entire body surface is covered by spines with no ultrastructural changes (Bar = 3 μm); **(A, panels 4 and 5)** Longer incubation (48 h) of control schistosomula in vehicle-containing medium shows further development and growth (panel 4, Bar = 50 μm) compared with panel 2; and a higher magnification (panel 5, Bar = 3 μm) confirms that the surface is covered by spines with no ultrastructural changes. **(A, panels 6 and 7)** Schistosomula micrographs at the same magnification (Bar = 30 μm) showing the ventral sucker (*vs*) of typical schistosomula maintained for 16 h in culture medium (panel 6) or further incubated for 24 h in culture medium plus 20 μM GSK343 (panel 7); after 24 h in the presence of GSK343 the schistosomulum showed no apparent development or change in size, with an extremely contracted body region and the presence of numerous folds and small blisters (panel 7). **(A, panel 8)** Higher magnification of schistosomulum from panel 7 is an example showing that the teguments of parasites exposed to 20 μM GSK343 for 24 h have a high degree of changes including peeling and blister formation (Bar = 3 μm). **(A, panels 9 and 10)** Longer incubation of schistosomula (48 h) in culture medium plus 20 μM GSK343 showed no further development (panel 9) compared with the initial schistosomulum (panel 6), with the presence of a greater number of blisters when compared with panel 7; panel 10 shows a higher magnification of the schistosomulum from panel 9, with intense peeling of tegument and loss of numerous spines (Bar = 5 μm). (**B)** Total length of schistosomula incubated with vehicle (0.1% DMSO) or with 20 μM GSK343 for 24 and 48h. Mean ± SD from three replicate experiments, each with 20 schistosomula. (**C)** Increase in caspases 3/7 activity after 48 h treatment with 20 μM GSK343. Mean ± SD from three replicate experiments, each with 20,000 schistosomula. **(D, panels 1 to 4)** TUNEL assay of schistosomula incubated for 72 h with 0.1% DMSO (panels 1 and 2) or with 20μM GSK343 (panels 3 and 4). Green parasites indicate DNA fragmentation. In panel 1, DAPI nuclear DNA staining of control parasites (0.1% DMSO). In panel 2, TUNEL staining of control parasites from panel 1 (0.1% DMSO). In panel 3, DAPI nuclear DNA staining of GSK343 treated parasites. In panel 4, TUNEL staining of GSK343 treated parasites from panel 3. (*vs*), ventral sucker; (*s)*, spines; (*b*), blisters. ***p < 0.001.

Schistosomula mechanically transformed from cercariae were incubated for 16 h in culture medium and micrographs of typical schistosomula were obtained (Fig 5A, panels 1 and 6). At this time vehicle (0.1% DMSO) was added to control schistosomula, parasites were further incubated and normal development was documented at 24 h (Fig 5A, panels 2 and 3) and at 48 h (Fig 5A, panels 4 and 5). In contrast, when GSK343 was present in the medium, no apparent development or change in size was observed (Fig 5A, panels 7 and 9) compared with schistosomula pre-treatment (Fig 5A, panel 6). Deterioration of the tegument was observed at higher magnification (Fig 5A, panels 8 and 10), with the presence of many surface blisters, intense contraction and loss of numerous spines that covered the whole body of the parasite. Fig 5B shows that while the average length of control schistosomula significantly increased 1.4-fold in the period from 24 to 48 h on incubation with 0.1% DMSO (110 μm / 79 μm = 1.4), the average length of schistosomula treated with 20 μM GSK343 significantly increased only 1.1-fold (74 μm / 67 μm = 1.1) during the same period of time (Fig 5B), resulting in a significantly 4-fold lower rate of growth (p < 0.001) and in significantly smaller treated schistosomula at 48 h incubation (average length 74 μm) compared with controls (average length 110 μm) (p < 0.001).

Exposure of schistosomula for 48 h to 20 μM GSK343 significantly increased caspase 3/7 activity by 17-fold, when compared with control parasites (Fig 5C). Caspases 3 and 7 are activated in both the extrinsic and intrinsic apoptosis pathways [51], and their activity was also increased after treatment of schistosomes with the HDAC inhibitor TSA [12]. Additionally, the capacity of GSK343 to induce apoptosis in schistosomula was measured using a TUNEL assay to detect DNA double strand breaks in schistosomula treated with 20 μM GSK343 for 72 h. The results indicate that this inhibitor induced fragmentation of DNA, with specific green staining clearly observed along the entire worm (Fig 5D, panel 4). No auto fluorescence was detected, as can be seen by the absence of green coloring in the control (0.1% DMSO) worms observed under UV light (Fig 5D, panel 2).

**Fig 6:**
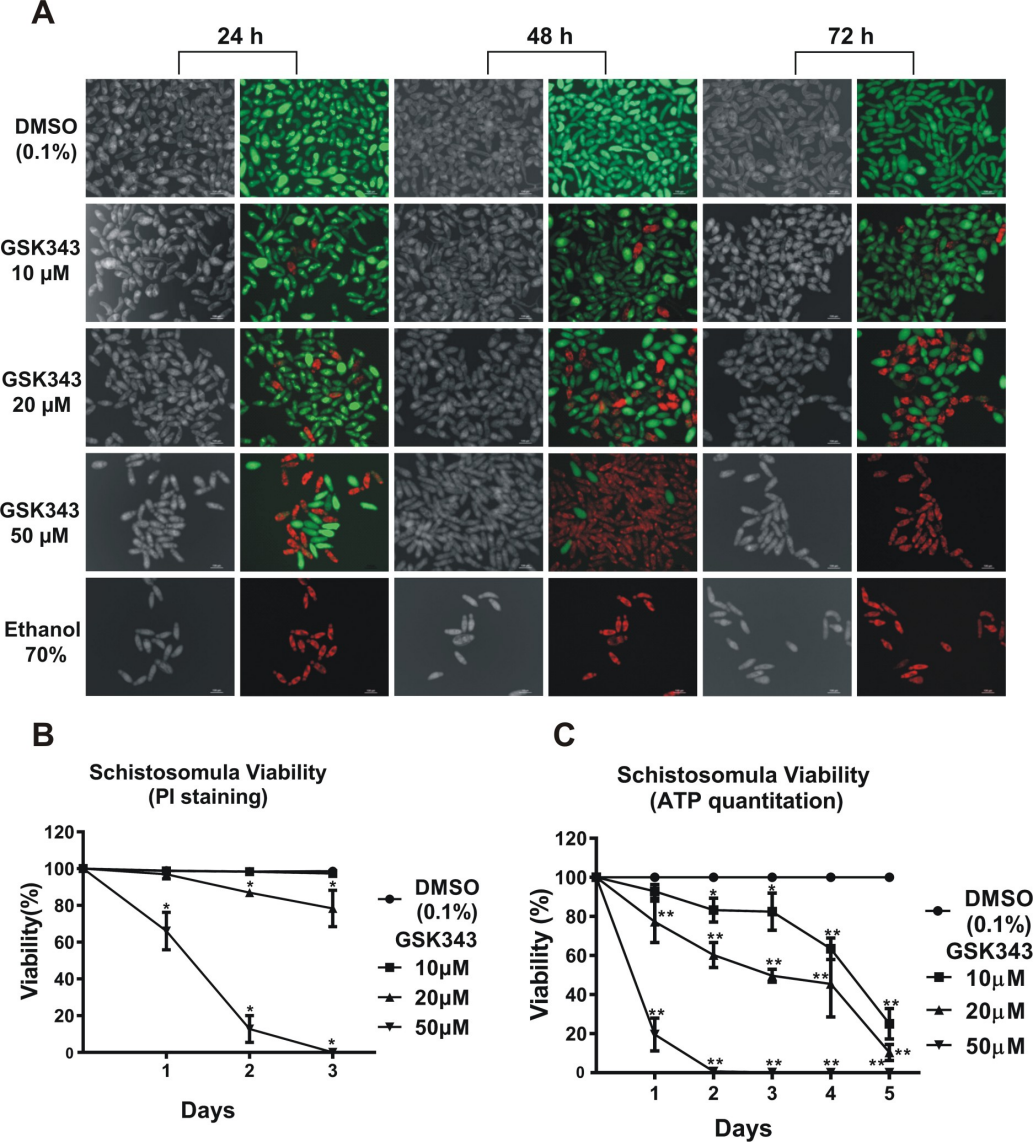
Quantitation of the decrease in viability of schistosomula caused by GSK343 treatment at different concentrations and incubation times. **(A)** Schistosomula treated with the indicated concentrations of GSK343 or with vehicle (0.1% DMSO) for 24, 48 and 72 h were visualized by staining with propidium iodide (marker of dead cells; 572 nm emission filter microscope) and with fluorescein diacetate (marker of living cells; 492 nm emission filter microscope). The bottom row shows a positive control, namely exposure to 70% ethanol, which kills all parasites. For each time point (indicated at the top), the left panel shows a light microscopy image and the right panel shows the image of the same field with differential fluorescence detection of PI-positive dead and FDA-positive live schistosomula by superimposition of 536nm and 494nm epifluorescence spectra. **(B)** Quantitation of viability of treated schistosomula. Percentage of viable schistosomula (non-stained with propidium iodide) over three days of treatment. For each condition tested, about 3600 schistosomula were used, divided into four biological replicates and three time points analyzed. Mean ± SD from four replicate experiments. **(C)** ATP quantitation using a luminescent assay to assess schistosomula survival under GSK343 exposure. Schistosomula (100-120/well) were incubated with the indicated concentrations of GSK343 or with vehicle (0.1% DMSO) for up to 5 days. The viability was expressed as % the luminescence values relative to the control (DMSO). Mean ± SD from three replicate experiments. *p < 0.05; **p < 0.01 (two-way ANOVA).

Fluorescein diacetate (FDA) [29] and propidium iodide (PI) [28] were used to differentially quantitate viability of schistosomula at 24, 48 and 72 h incubation (Fig 6A) in the presence of vehicle or of 10 to 50 μM GSK343. Under fluorescence microscopy, death of schistosomula in the presence of GSK343 was detected by a red fluorescence signal from PI (536 nm emission microscope filter). Viability was quantified in schistosomula living cells, where FDA is converted into charged fluorescein by the parasite esterase activity, staining the schistosomula with a green fluorescence signal (492 nm emission microscope filter) (Fig 6A).

Living green-stained schistosomula were counted at each GSK343 concentration and time. Percentage viability was determined with respect to the total number of parasites present in the well, as checked and counted using light optical microscopy (see Fig 6A, gray images), and plotted as shown in Fig 6B. Incubation with vehicle (0.1% DMSO) for up to 72 h showed no reduction of parasite viability (Fig 6A, upper panels and 6B), whereas 20 μM GSK343 proved to be a sub-lethal concentration because there was less than 20% decrease in schistosomula viability after 2 days of incubation with GSK343 (Fig 6B). On the other hand, ATP quantitation (Fig 6C) in treated schistosomula showed that the parasites were stressed by the compound, and a reduction of 40% in total ATP content was observed after 2 days of incubation at 20 μM GSK343. It was observed that at longer incubation times for up to 5 days, all tested concentrations of GSK343 caused a 70–100% decrease in viability as measured by ATP content.

### Profile of post-translational modifications in histones of *S*. *mansoni* treated with GSK343

To detect and generate the profile of changes in histone marks triggered by GSK343 in *S*. *mansoni*, the nuclear extracts of adult worms and schistosomula treated with this inhibitor of EZH2 histone H3K27 methyl-transferase were analyzed on western blots developed with a monoclonal antibody specific for the H3K27me3 post-translational modification (PTM), which is related to chromatin condensation and repression of transcription. Four other histone marks, not directly related to EZH2 histone activity, were assayed in order to document the profile of histone marks in the treated parasites.

**Fig 7:**
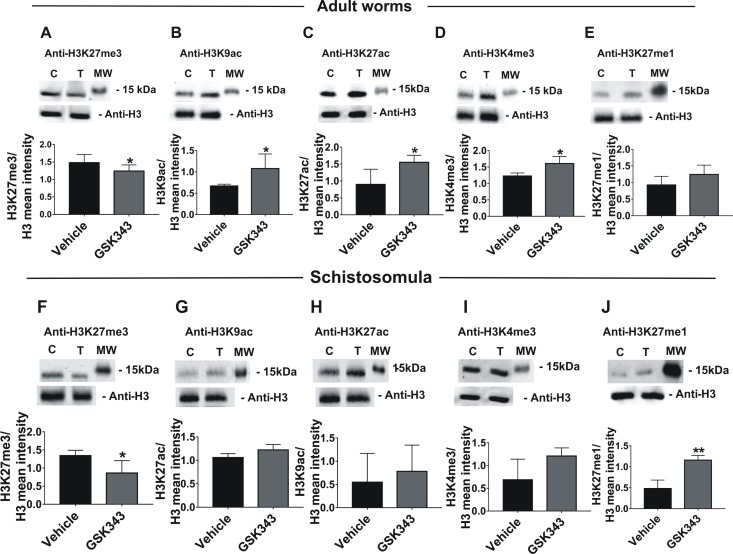
Histone H3 post-translational modification profile in *S*. *mansoni* after treatment with GSK343. Five different histone marks in adult worms **(A to E)** and in schistosomula **(F to J)** were detected and quantified by western blotting with **(A and F)** anti-H3K27me3, **(B and G)** anti-H3K9ac, **(C and H)** anti-H3K27ac, **(D and I)** anti-H3K4me3, **(E and J)** anti-H3K27me1 antibodies. Each panel consists of two parts: the upper part shows the lanes of a typical western blot of nuclear extract from untreated control parasites (C) or parasites treated for 48 h with 20 μM GSK343 (T) which were developed either with the specific antibody against the indicated histone mark or with an antibody anti-H3, used as a sample loading normalizer. A molecular weight marker protein (MW, 15 kDa) is indicated. The lower part of each panel shows the mean intensity of the bands for three biological replicates, obtained by extracting the intensity values of scanned images; for each sample, the intensity of the modified histone band was normalized by the intensity of histone H3. Mean ± SD is shown; t-test was applied and statistically significant p-values are indicated: *p < 0.05; **p < 0.01.

A significant decrease in the level of the H3K27me3 mark was quantitated in adult worms (Fig 7A) and in schistosomula (Fig 7F), treated with GSK343 for 48 h relative to the control (vehicle).

In adult worms the acetylated histone marks H3K9ac and H3K27ac (Fig 7B and 7C) were concomitantly and significantly increased in relation to the control samples (vehicle) in three biological replicates. In schistosomula, on the other hand, these marks showed a slight tendency to increase, although these changes were not statistically significant (Fig 7G and 7H). Two more histone marks were analyzed, namely H3K4me3 and H3K27me1; in adult worms there was a significant increase only in H3K4me3 (Fig 7D and 7E), while in schistosomula there was a significant increase only in H3K27me1 (Fig 7I and 7J).

With the above panel of histone PTMs, it is possible to conclude that the decrease in the H3K27me3 mark caused by GSK343 treatment probably affected the chromatin architecture [52], thus triggering a compensatory reprogramming and fine-tuning of the parasite gene expression that possibly resulted from a change in the balance between H3K27me3 and other histone marks such as H3K9ac, H3K27ac, H3K4me3 and H3K27me1 [52–54], which are known to be related to activation of gene transcription.

### GSK343 causes large-scale gene expression inhibition in adult worms and schistosomula

To explore the effect of GSK343 on gene expression, adult worms and schistosomula were exposed *in vitro* to the compound or to vehicle (DMSO), and large-scale gene expression changes were assessed by RNA-Seq. It is known that using different RNA-Seq statistical analyses tools can affect both the number of differentially expressed genes (DEGs) and the altered processes or functions that are detected [55,56]. Therefore, we used three different pipelines, namely Kallisto + Sleuth [41], SailFish + EdgeR [43] and Tuxedo Tools (including Cuffdiff) [40], and looked for a core set of consensus DEGs among them, in order to increase the accuracy of the list of DEGs identified. An example is given in S1 Text, Fig A(i), left panel, which shows the Venn diagram with 585 consensus DEGs (S2 Table) that were detected as downregulated in adult female parasites in the intersection of these three statistical analyses pipelines. It is apparent that between 56 and 245 genes were detected by only one pipeline, and these genes were not further analyzed. In all other analyses of females, males, and schistosomula a similar pattern was obtained, with a core set of consensus genes being considerably larger than the genes exclusively detected by only one pipeline (S1 Text, Figs A(i), A(ii) and A(iii)). The lists of significantly downregulated (S2 Table) and upregulated (S3 Table) consensus genes in treated females, as well as the significantly downregulated (S4 Table) and upregulated (S5 Table) consensus genes in treated males are given in the Supplementary Information.

Interestingly, the effect of GSK343 on adult worms was sex-specific (S1 Text, Figs A(i) and A(ii)): only a small number of genes was detected in common as downregulated between the two sexes (45 genes, corresponding to 7.7% of all downregulated genes in females and 7.9% of all downregulated genes in males). Similarly, only 82 genes were detected in common as upregulated between females and males, corresponding to 18.3% of all upregulated genes in females and 15.1% of all upregulated genes in males (S1 Text, Figs A(i) and A(ii)). It is noteworthy that in schistosomula we detected a smaller number of consensus DEGs when compared with adult worms, namely 143 downregulated (S6 Table) and only 29 (S7 Table) upregulated genes (S1 Text, Fig A(iii)).

**Fig 8:**
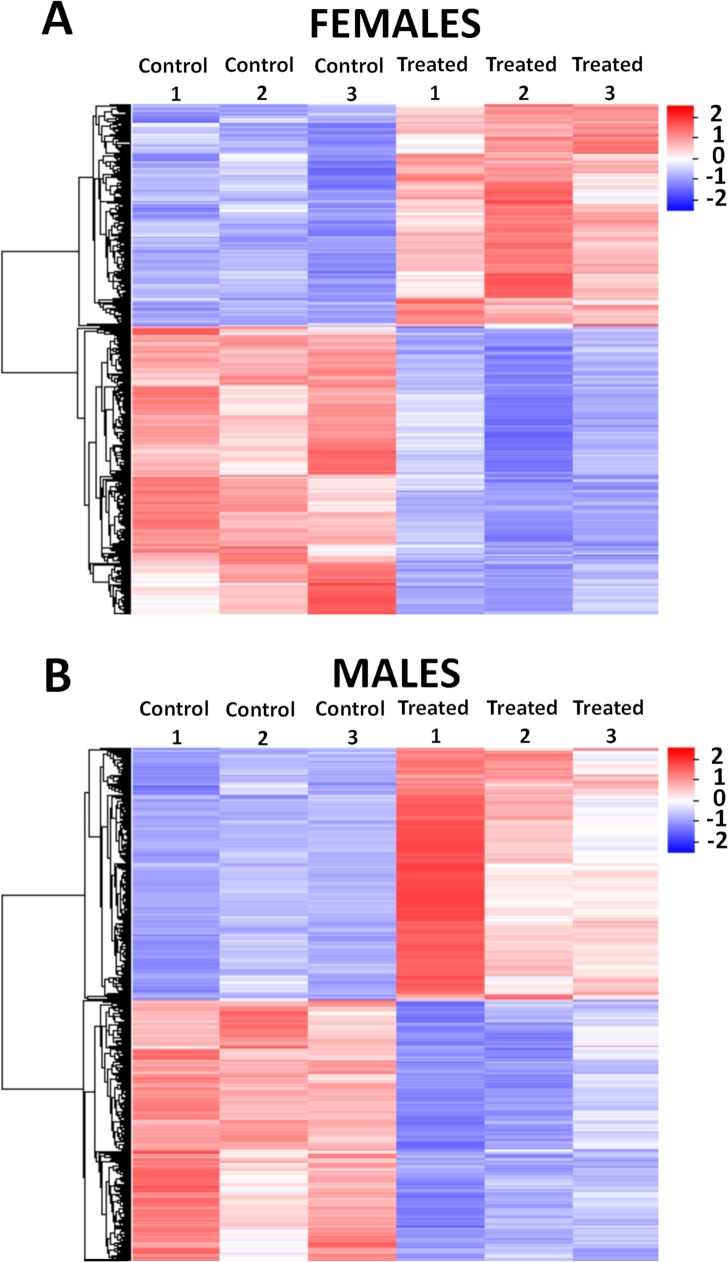
Heatmap of differentially expressed genes detected by RNA-seq in adult *S*. *mansoni* female and male worms treated with GSK343. The heatmaps show the hierarchical clustering of differentially expressed genes (lines) in three biological replicates (columns) of female **(A)** and male **(B)** adult worm samples, either for controls or for treated parasites, as indicated at the top of the heatmaps. Parasites were exposed for 48 h *in vitro* to vehicle (control) or to 20 μM GSK343. Gene expression levels were measured by RNA-seq and are shown as Z-scores, which are the number of standard deviations below (blue, downregulated) or above (red, upregulated) the mean expression value among treated and control samples for each gene; the expression level Z-scores are color-coded as indicated on the scale at the bottom.

**Fig 9:**
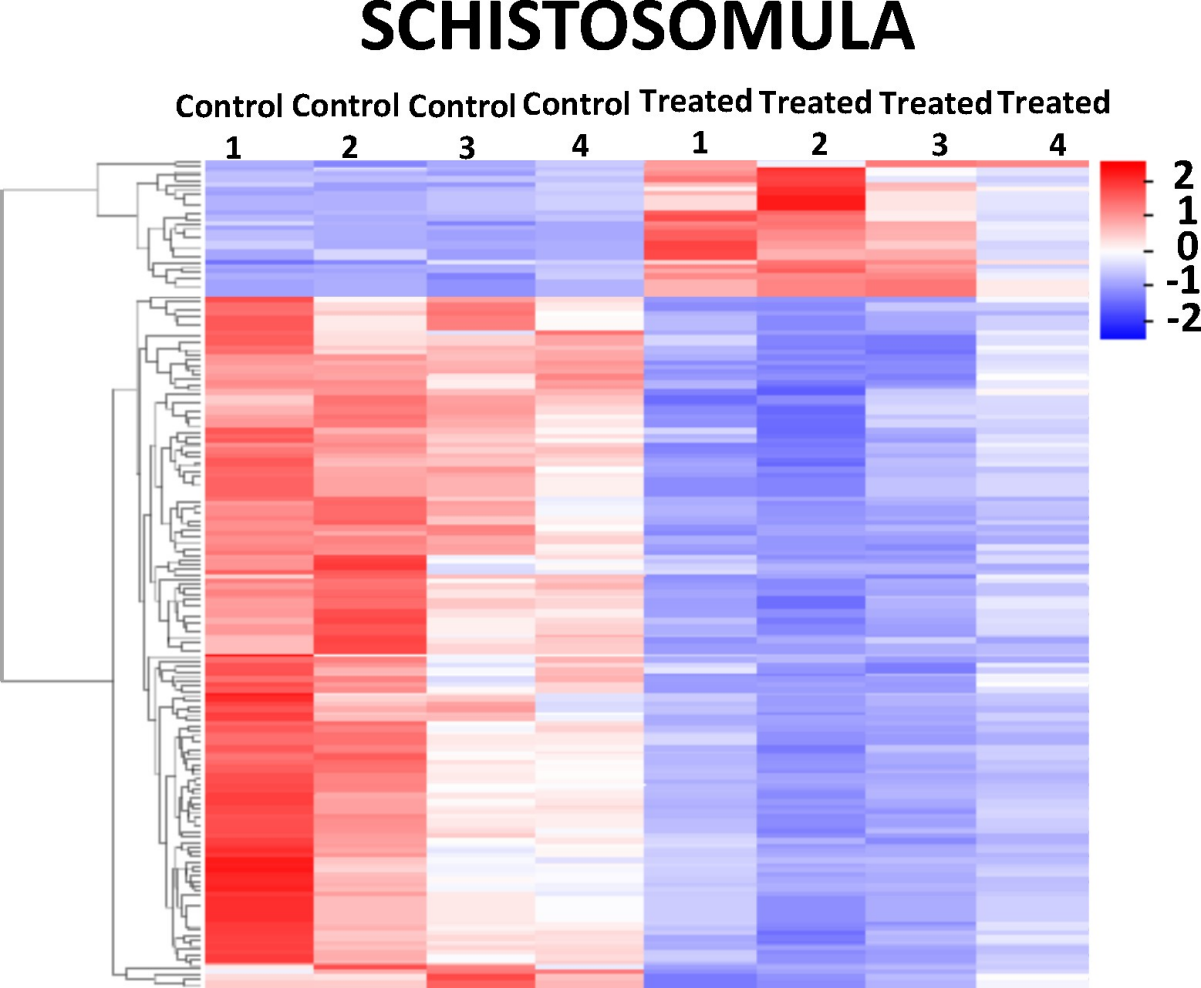
Heatmap of differentially expressed genes detected by RNA-seq in schistosomula treated with GSK343. The heatmap shows the hierarchical clustering of differentially expressed genes (lines) in four biological replicates (columns) of schistosomula samples, either for controls or for treated parasites, as indicated at the top of the heatmap. Parasites were exposed for 48 h *in vitro* to vehicle (control) or to 20 μM GSK343. Gene expression levels were measured by RNA-seq and are shown as Z-scores, which are the number of standard deviations below (blue, downregulated) or above (red, upregulated) the mean expression value among treated and control samples for each gene; the expression level Z-scores are color-coded as indicated on the scale at the bottom.

The consensus set of DEGs can be better visualized in the heatmaps of Figs 8and 9, where a marked difference in the gene expression levels can be seen between control and treated samples, in females (Fig 8A), males (Fig 8B) and schistosomula (Fig 9), for both upregulated (red) and downregulated (blue) genes.

**Fig 10:**
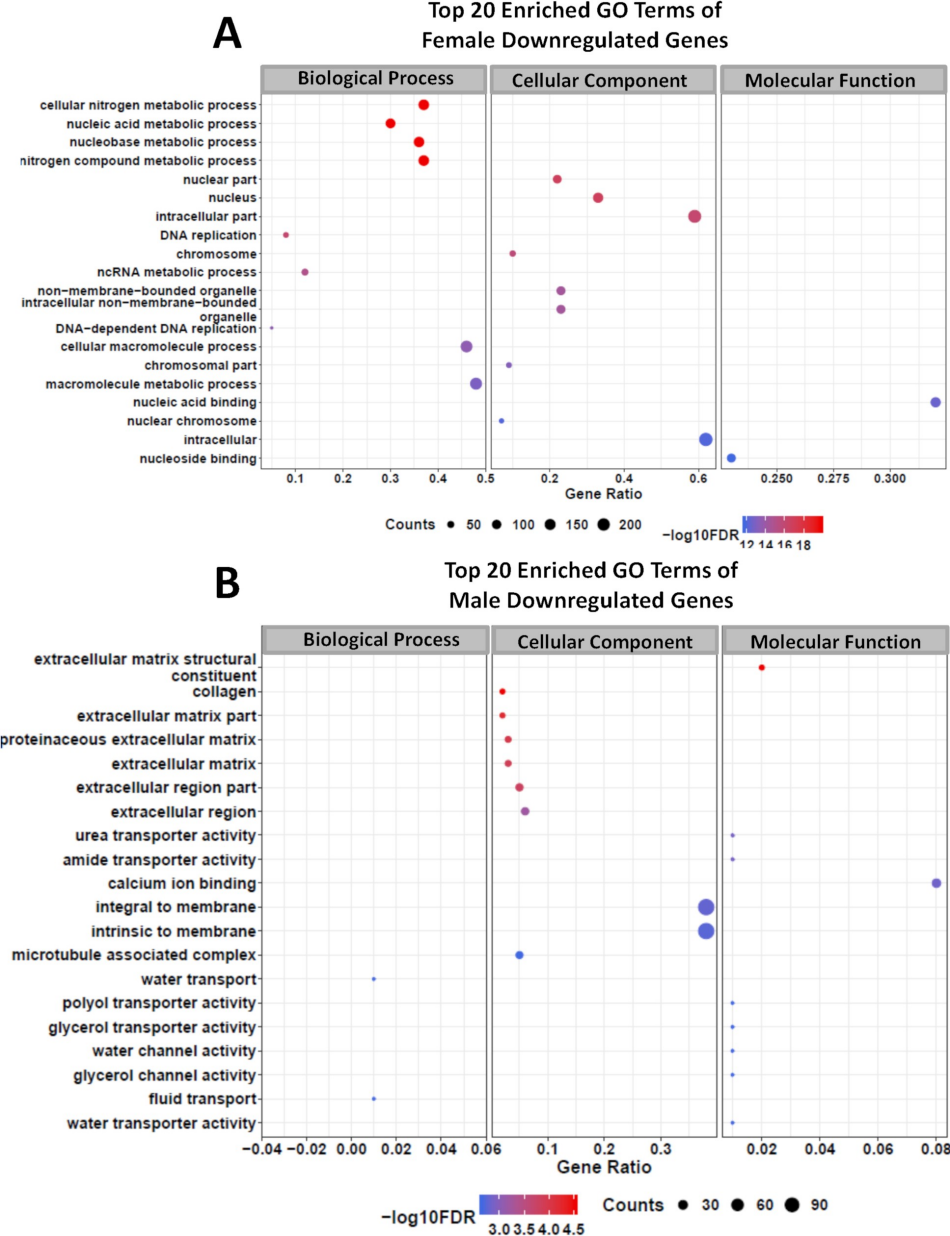
Gene Ontology terms enrichment analysis of downregulated genes detected by RNA-seq in adult *S*. *mansoni* female and male worms treated with GSK343. Top 20 enriched GO terms for differentially expressed downregulated genes in female **(A)** and in male **(B)** adult worms. The three major GO term categories, namely Biological Process, Cellular Component and Molecular Function are separately represented in each panel. The size of the circles is proportional to the number of genes in each significantly enriched category, as indicated at the lower part scales; the colors show the statistical significance of the enrichment, as indicated by the -log10 FDR values that appear in the color-coded scales at the bottom. A GO enrichment significance cutoff of FDR ≤ 0.05 was used.

**Fig 11:**
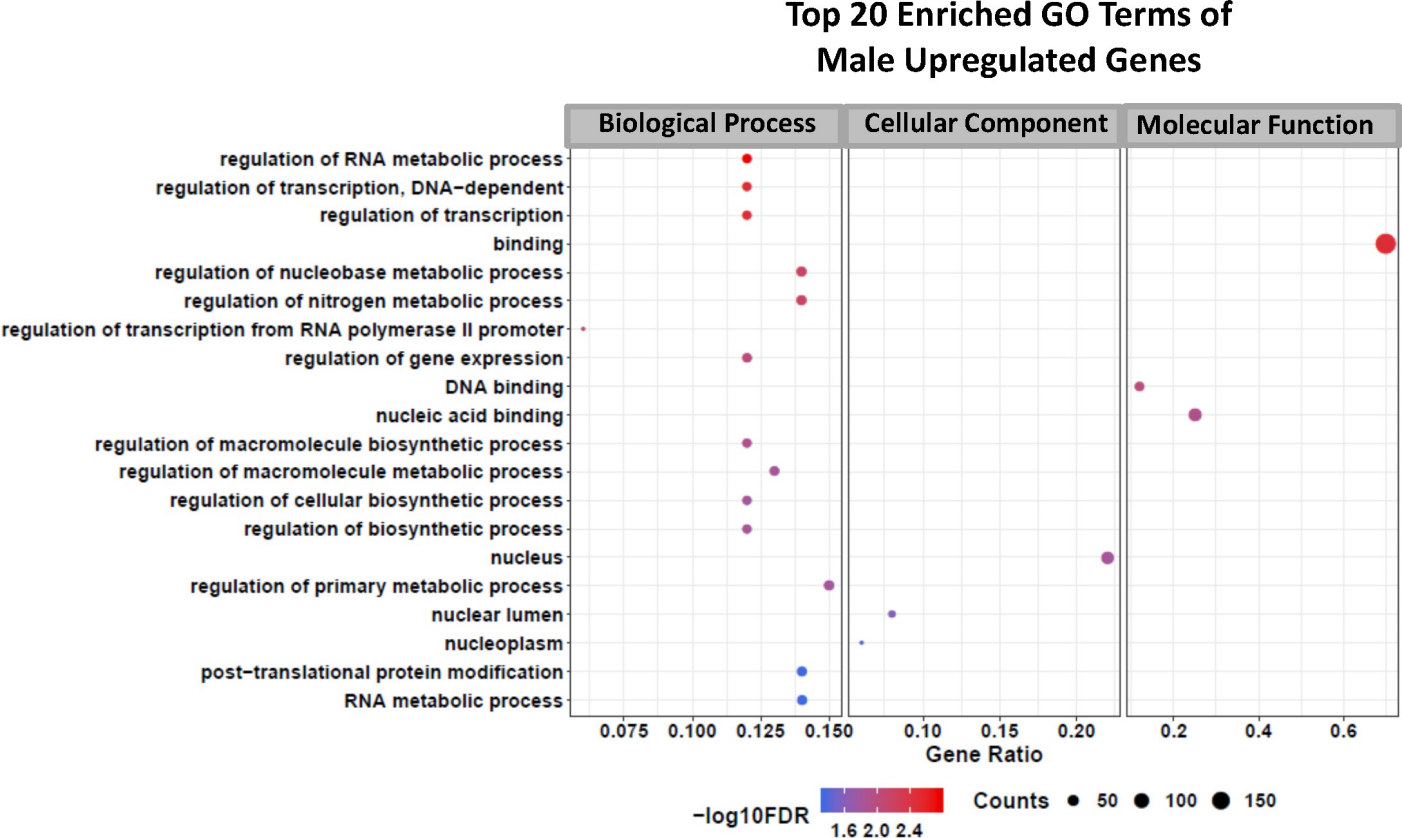
Gene Ontology terms enrichment analysis of upregulated genes detected by RNA-seq in adult *S*. *mansoni* male worms treated with GSK343. Top 20 enriched GO terms for differentially expressed upregulated genes in male adult worms. The three major GO term categories, namely Biological Process, Cellular Component and Molecular Function are separately represented in each panel. The size of the circles is proportional to the number of genes in each significantly enriched category, as indicated at the lower part scales; the colors show the statistical significance of the enrichment, as indicated by the -log10 FDR values that appear in the color-coded scales at the bottom. A GO enrichment significance cutoff of FDR ≤ 0.05 was used; no significantly enriched GO terms were found among the female upregulated genes.

With the lists of female and male differentially expressed genes at hand, a gene ontology (GO) analysis was performed, and Figs 10 and 11 show the top 20 GO enriched terms for downregulated genes in female (Fig 10A) and male worms (Fig 10B) as well as the enriched terms for upregulated genes in males (Fig 11). No significantly enriched GO terms (FDR ≤ 0.05) were detected among the upregulated genes in females. GSK343 caused alterations in genes related to various metabolic pathways, which are different between females and males, as expected from the minimal overlap of DEGs (S1 Text, Figs A(i) and A(ii)).

**Fig 12:**
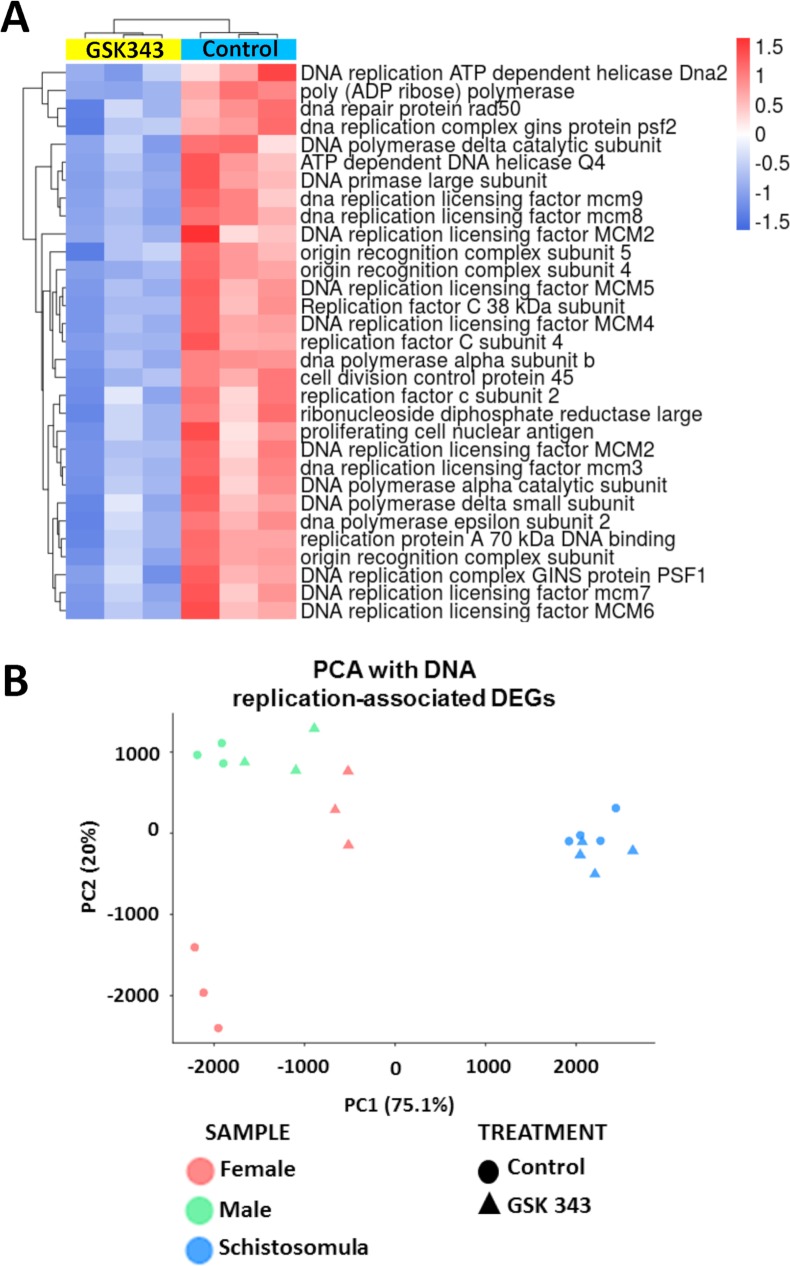
Genes related to DNA replication that were downregulated in GSK343 treated female adult worms. **(A)** Heatmap of 32 genes related to DNA replication that were downregulated in treated females. Genes are shown on the lines, and three biological replicates are shown in the columns, for control (turquoise bar at the top of columns) or GSK343-treated female samples (yellow bar at the top of columns). Parasites were exposed for 48 h *in vitro* to vehicle (control) or to 20 μM GSK343. Gene expression levels were measured by RNA-seq and are shown as Z-scores, which are the number of standard deviations below (blue, downregulated) or above (red, upregulated) the mean expression value among treated and control samples for each gene; the expression level Z-scores are color-coded as indicated on the scales at the bottom. The heatmap was obtained with unsupervised clustering of samples and genes. **(B)** Principal component analysis (PCA) of expression of the 32 genes from (A) that were measured by RNA-seq in control and treated females, males and schistosomula, as indicated by the legend at the bottom.

In female worms, the GSK343-induced downregulated genes that belong to enriched Molecular Function GO terms are related to nucleic acid and nucleoside binding (Fig 10A). Downregulated genes belonging to enriched Biological Process GO terms are essentially related to nucleic acid metabolic processes, and more specifically the DNA replication pathway and the ncRNA metabolic process (Fig 10A). A heatmap representing all DNA replication genes that were detected as downregulated in GSK343 treated females (32 genes) is shown in Fig 12A; the list of these genes with Smp ID numbers and gene descriptions is given in S8 Table.

Interestingly, a principal component analysis (PCA) using the expression levels of this set of 32 genes in females, males and schistosomula (Fig 12B) revealed that females treated with GSK343 have an expression profile of the replication machinery genes quite different from that of untreated females and very similar to those of treated and untreated male worms, suggesting that the active DNA replication machinery in control females is involved with embryonic division and oviposition. The schistosomula expression profile of DNA replication genes is very different from that of adult worms (Fig 12B).

**Fig 13:**
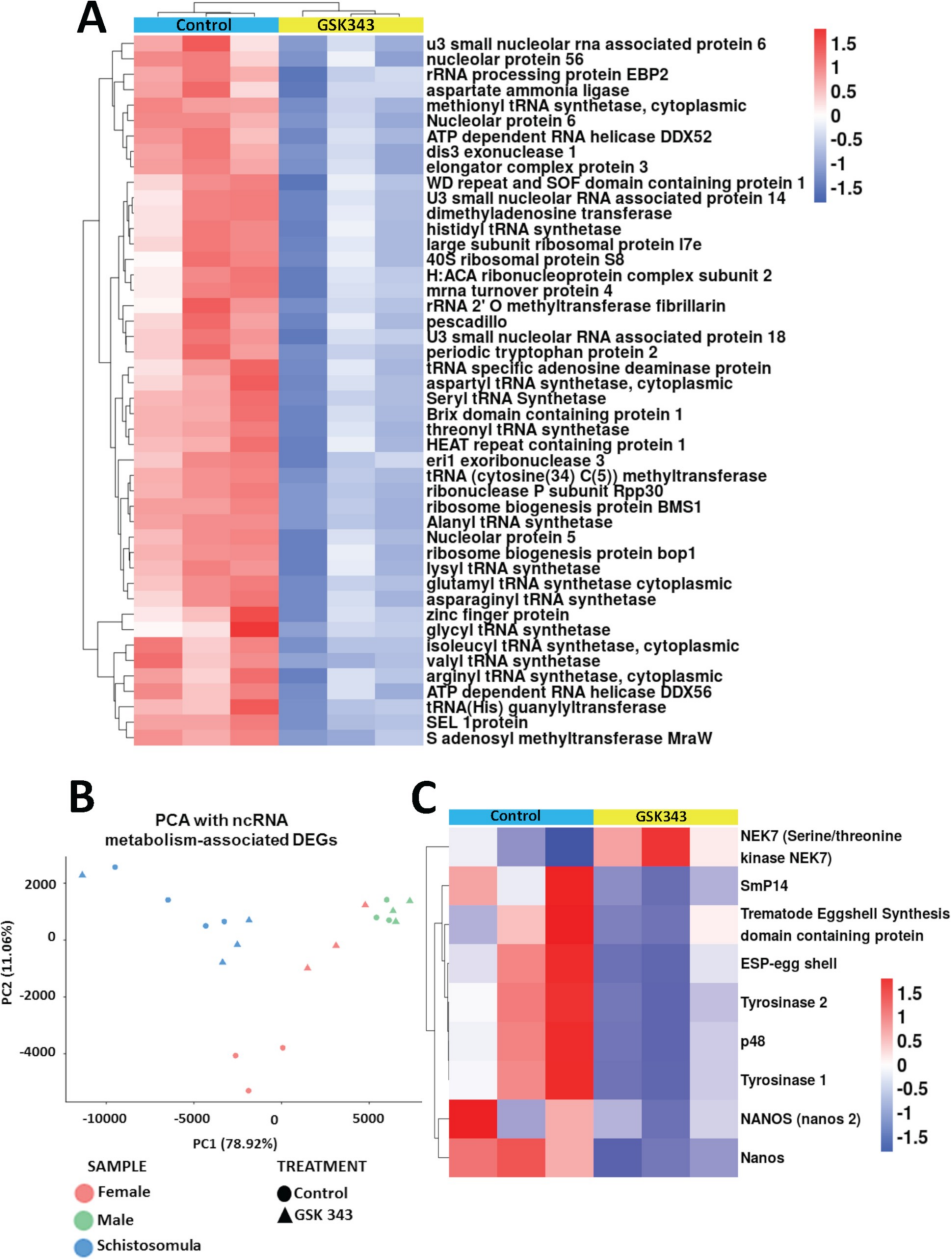
Genes related to ncRNA metabolism, egg biosynthesis and cell differentiation that were downregulated in GSK343 treated female adult worms. **(A)** Heatmap of 51 genes related to ncRNA metabolism that were downregulated in treated females. The heatmap was obtained with unsupervised clustering of samples and genes. **(B)** Principle component analysis (PCA) of expression of the 51 genes from (A) that were measured by RNA-seq in control and treated females, males and schistosomula, as indicated by the legend at the bottom. **(C)** Heatmap of 9 selected genes, related to egg biosynthesis and cell differentiation, whose expression was significantly affected in GSK343-treated females. Heatmap obtained with unsupervised clustering of genes. In the heatmaps of (A) and (C), genes are shown on the lines, and three biological replicates are shown in the columns, for control (turquoise bar at the top of columns) or GSK343-treated female samples (yellow bar at the top of columns). Parasites were exposed for 48 h *in vitro* to vehicle (control) or to 20 μM GSK343. Gene expression levels were measured by RNA-seq and are shown as Z-scores, which are the number of standard deviations below (blue, downregulated) or above (red, upregulated) the mean expression value among treated and control samples for each gene; the expression level Z-scores are color-coded as indicated on the scales at the bottom.

The heatmap of ncRNA metabolic process genes that were detected as downregulated in GSK343 treated females (Fig 13A, 51 genes) shows that these genes essentially encode rRNA proteins and tRNA processing proteins as well as proteins involved in translation; the list of these genes with Smp ID numbers and gene descriptions is given in S8 Table. Again, a PCA using the expression levels of these 51 genes involved in ncRNA metabolic processing (Fig 13B) shows that females treated with GSK343 have an expression profile of ncRNA metabolic process genes very similar to that of treated and untreated male worms, possibly indicating that, as is the case for the active DNA replication machinery, the active ncRNA metabolism in control females is related to protein synthesis involved in embryonic division and oviposition.

Considering that the oviposition pathways in *S*. *mansoni* are not annotated in the GO database, and that correct eggshell formation is essential for embryonic cell differentiation and development, we searched for genes described in the literature as related to egg formation [15,57]. Fig 13C shows the expression levels of genes encoding four eggshell proteins, two tyrosinases, one serine/threonine kinase, all involved in egg production, and nanos-1 and -2, related to egg production and cell differentiation [58,59]; the list of these genes with Smp ID numbers and gene descriptions is given in S8 Table. All genes except NEK7 serine/threonine kinase were downregulated by GSK343 treatment (Fig 13C).

Accordingly, we have searched for genes described in the literature as related to the tegument and gut [60–62] and we found 19 gut genes downregulated in males exposed to GSK343, including nine genes from the microexon gene (MEG) family (MEG-1, two MEG-4, MEG-6, two MEG-8, MEG-9, MEG-11, MEG-14), one annexin, one tetraspanin family member, two phospholipase genes, two genes encoding saposin domain containing proteins, two cathepsin peptidases, the phosphatidylcholine sterol acyltransferase gene and a gene encoding a 25 kDa integral membrane protein (S9 Table). In females, 5 gut genes were downregulated including one from the MEG-4 family, one encoding the venom allergen-like 7 protein (VAL 7), two tRNA synthetases and one phospholipase gene (S9 Table). Schistosomula early gut genes expression was as well extensively affected by GSK343 exposure, with 19 downregulated genes, including genes encoding MEGs, cathepsins, saposins, VAL 7, tetraspanins, phospholipase and palmitoyl protein thioesterase (S9 Table).

Interestingly, only 2 gut genes were upregulated in GSK343-exposed males, namely one annexin and one zinc finger DHHC type gene, whereas in females only 6 gut genes were upregulated, namely three tetraspanins, one annexin and two palmitoyl protein thioesterase genes; no gut genes were upregulated in schistosomula (S9 Table).

An additional 16 genes related to the tegument [61,62] were found downregulated by GSK343 exposure of male worms, namely the genes encoding aquaporin-9, dysferlin1, sodium coupled neutral amino acid transporter, Na/K ATPase beta subunit, calcium transporting ATPase, cation transporting ATPase, MDR transporter, Sm200 surface glycoprotein, seven different tegument-allergen-like proteins including Sm13 and a hypothetical protein (Smp_081920) (S9 Table). In females, 10 genes related to the tegument were found downregulated, including the genes encoding aquaporin-9, Na/K ATPase alpha and beta subunits, two cation-transporting ATPases, three MDR transporters and two tegument-allergen-like proteins including Sm13 (S9 Table). In schistosomula, 4 genes associated with the tegument were found downregulated, encoding a sodium/chloride dependent transporter, a tetraspanin, the Sm200 surface glycoprotein and a tegument-allergen-like protein (S9 Table).

Again, much fewer genes associated with the tegument were found upregulated in GSK343-exposed parasites compared with the downregulated, namely 4 genes in males, encoding a transient receptor potential cation channel, two MDR transporters and acetylcholinesterase; in females, 5 genes were found, encoding acetylcholinesterase, a RAB18 RAS oncogene family member, carbonic anhydrase, major tegumental antigen Sm15 and a hypothetical protein (Smp_105220) (S9 Table). No upregulated genes associated with the tegument were found in GSK343-exposed schistosomula.

In male worms, GSK343 treatment caused downregulation of genes belonging to enriched Molecular Function GO terms related to the membrane constituents and transport of water, glycerol and urea (Fig 10B). Genes related to the structural constituents of the extracellular matrix were downregulated and belong to a number of enriched Cellular Component and Molecular Function GO terms (Fig 10B). Upregulated genes in males belong to enriched Biological Process GO terms related to regulation of RNA metabolic process and regulation of transcription (Fig 11).

**Fig 14:**
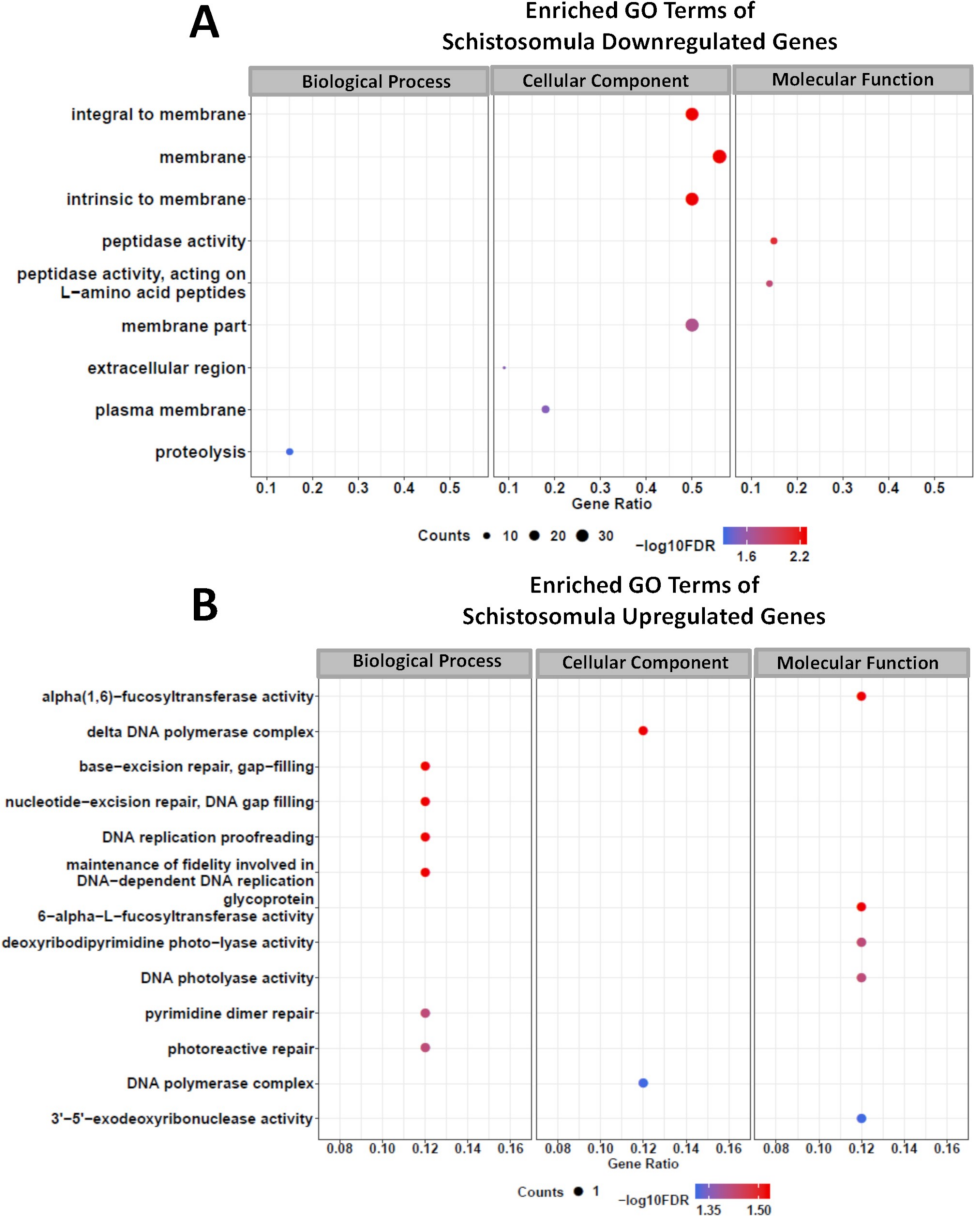
Gene Ontology terms enrichment analysis of differentially expressed genes detected by RNA-seq in schistosomula treated with GSK343. Enriched GO terms for differentially expressed genes in schistosomula, separated by downregulated genes **(A)** and upregulated genes **(B)**. The three major GO term categories, namely Biological Process, Cellular Component and Molecular Function are separately represented in each panel. The size of the circles is proportional to the number of genes in each significantly enriched category, as indicated at the lower part scales; the colors show the statistical significance of the enrichment, as indicated by the -log10 FDR values that appear in the color-coded scales at the bottom. A GO enrichment significance cutoff of FDR ≤ 0.05 was used.

In schistosomula treated with GSK343, the set of downregulated genes that belong to enriched Molecular Function GO terms are related to peptidase activity and those belonging to Biological Process GO terms are related to proteolysis (Fig 14A). Also downregulated were genes belonging to enriched Cellular Component GO terms related to membrane constituents (Fig 14A). Of note, very few genes were upregulated in schistosomula treated with GSK343 (29 genes) (Fig 9) and only one gene was present in each of the enriched GO terms that have been detected (Fig 14B), which include a number of Biological Process GO terms related to DNA repair and DNA replication proof-reading (Fig 14B).

### Validation by quantitative PCR of genes detected by RNA-Seq as affected by GSK343

A set of sixteen differentially expressed genes with statistically significant change of expression was selected for RT-qPCR validation of the RNA-Seq results (S1 Text, Fig B). The selection was based on the following criteria: genes downregulated in treated females (S1 Text, Fig B(i)) or schistosomula (S1 Text, Fig B(ii)) that are involved in egg formation, and participate in cell differentiation, signaling pathways, membrane constituents, PRC2 complex and interaction between the mating pair.

Tested genes involved in egg biosynthesis were all confirmed by qPCR as downregulated in treated females (S1 Text, Fig B(i)); they are p48, p14, ESP (egg shell protein), Tyrosinases 1 and 2. The other genes confirmed by qPCR as downregulated in GSK343 treated-females were the nanos gene, which is involved in vitelline cell differentiation [58], the EED gene, which encodes a PRC2 component that is required for regulation of the catalytic activity of the EZH2 enzyme, and EEFB1 (eukaryotic translation elongation factor 1 beta) (S1 Text, Fig B(i)).

In schistosomula, genes that were validated by qPCR as downregulated (S1 Text, Fig B(ii)) included MEG-4 and Val7, present in the digestive tract of the different developmental forms of *S*. *mansoni* [47,63]. Other validated genes encode LDL receptor, FA binding protein and Glucose transport protein (Smp_105410), components of the tegument membrane. Genes encoding the gynecophoral canal protein, involved in mating, the Dnase II protein, involved in degradation of exogenous DNA for cell homeostasis, and the FGFR substrate 3, a ligand of the FGFR that stimulates the Ras pathway, were also validated by qPCR as significantly downregulated in treated schistosomula (S1 Text, Fig B(ii)).

### Proteome analysis of *S*. *mansoni* adult worms treated with GSK343

Given the fact that RNA-seq analyses suggested that GSK343 treatment of parasites caused a marked decrease in the expression of 51 genes belonging to the ncRNA GO term and involved with protein synthesis pathways (see above), we decided to perform label-free quantitative mass spectrometry-based proteomic analyses to identify and quantitate proteins differentially abundant between treated and untreated parasites.

A total of 1136 proteins (S10 Table) were identified in total protein extracts of adult worms treated with GSK343 or of controls (0.1% DMSO). Among this set of proteins, 58 were significantly differentially abundant (S1 Text, Fig C), of which 24 of them (41%) were detected as less abundant in treated parasites compared with controls (S1 Text, Fig C). The list of proteins with the Smp ID number, gene product description and the LFQ values is given in S11 Table.

Interestingly, three proteins related to the protein synthesis pathways were detected as less abundant in treated worms compared with controls, namely Ribosomal protein S12 (Smp_060090), ATP dependent RNA helicase Ddx1 (Smp_163110) and Ribosomal protein l5 (Smp_200920) (S1 Text, Fig C). Two proteins that were found more abundant in treated parasites, namely calnexin (Smp_043150) and calreticulin (Smp_030370) (S1 Text, Fig C), specifically act to retain unfolded or unassembled N-linked glycoproteins in the endoplasmic reticulum [64].

Three additional proteins detected by LFQ MS as less abundant in treated worms (S1 Text, Fig C) caught our attention because they were downregulated as well in the RNA-Seq of schistosomula and adult worms. The first was epidermal growth factor receptor (Smp_035260), an important key mediator of cell communication during animal development and homeostasis; the second was fatty acid binding protein (Smp_046800), which is related to the trafficking of molecules and the formation of membranes; the third was tegument-allergen-like protein (Smp_086470) that may play a role in parasite–host interactions such as nutrient transport, environmental signal transduction, and evasion of host’s immune system [65,66]. Of note, an additional twenty proteins were uniquely detected (S12 Table) in the control samples (16 proteins) or in samples of treated adult worms (4 proteins).

Finally, staphylococcal nuclease domain containing-1 (SND1) (Smp_081570) was detected as less abundant in treated parasites (S1 Text, Fig C); SND1 is a very conserved multifaceted protein that participates in various intracellular processes, such as RNA interference, acting as a nuclease in the RNA-induced silencing complex (RISC), mRNA splicing and stability, and regulation of transcription acting as a co-activator [67].

## Discussion

In the present work we show that GSK343, a known inhibitor of human EZH2 [17], is a schistosomicidal compound, effectively acting *in vitro* against female and male adult worms. We have previously detected that GSK343 acted synergistically with TSA, a histone deacetylase (HDAC) inhibitor, to enhance the death of schistosomula *in vitro* [15]. Recently, Roquis et al. [68] have used GSK343 to show that histone H3K27 trimethylation is required for life cycle progression from miracidium to sporocyst. The LD50 for GSK343 was previously determined to be 24.5 μM [15], and here we confirmed that exposing adults and schistosomula for 24 h to 20 μM GSK343, a sub-lethal dose below the LD_50_, caused only a 10% decrease both in motility and in adult worm pairing. Notably, under this condition the compound already produced extensive morphological changes in the tegument of adults, and a significant reduction in egg size and oviposition. Also, a marked slow-down in schistosomula growth and development was observed. Similarly, juvenile schistosomula, which are resistant to praziquantel [23] and become sensitive when PZQ is combined with other compounds [22,29], had their tegument significantly affected by GSK343 after 48 h of exposure, although exhibiting a lesser decrease in viability as measured by ATP quantitation, when compared with adult worms. This is consistent with the finding that juvenile worms express higher levels of multidrug resistance-associated protein 1 (SmMRP1) compared with adult worms [69].

Interestingly, RNA-seq large-scale gene expression analyses of GSK343-treated adult females showed 32 genes with significantly downregulated expression that are related to DNA replication. It is noteworthy that TSA inhibition of histone deacetylases (HDACs) caused an opposite effect, namely a marked increase in gene expression of 21 genes involved in DNA replication [15]; it is apparent that the DNA replication program in *S*. *mansoni* is submitted to an extensive control by both histone trimethylation and deacetylation, which may be one factor that contributes to the previously reported synergy between TSA and GSK343 [15]. In addition, a pronounced downregulation in the expression of 51 genes related to ncRNA metabolism, such as genes encoding rRNA proteins and tRNA processing enzymes, was observed here, implying a possible reduction of the protein synthesis pathways in GSK343-treated females. In future studies it would be interesting to look at the GSK343 effect on the expression levels of noncoding genes such as tRNAs, by preparing RNA-seq libraries from ribosomal-RNA-depleted samples instead of from polyA+ RNA samples. It is interesting to note that label-free quantitative proteomic analyses showed that three ribosomal proteins are less abundant in total protein extracts from adult worms treated with GSK343, compared with controls.

An interesting characteristic of GSK343-treated females was that the overall gene expression profiles of DNA replication genes and of ncRNA metabolism genes were more correlated to the respective expression profiles of control and treated males, and quite different from control females. Recently, unpaired females, in which the gonads are not developed, were also shown to have an overall gene expression profile more correlated to that of males [70]. Because mature paired female schistosomes lay approximately 350 eggs per day [71], they must be metabolically more active than males in order to support oogenesis. The fact that DNA replication and ncRNA metabolism gene profiles of GSK343-treated paired females are similar to those of males suggests that GSK343 predominately inhibits DNA replication and protein synthesis of actively dividing embryos in the female egg-producing gonads. In this respect, we found that nanos, a gene that is highly transcribed in the vitellarium [58], was downregulated in GSK343-treated females. Because nanos is also a stem cell gene marker in *S*. *mansoni* somatic stem cells [72] and larvae [59] it should be interesting in a future work to test the possible effects of GKS343 treatment on stem cells, using the EdU labeling assay [72]. GSK343 produced a decrease in egg laying in the adult female worms, and eggs were deformed. It is unlikely that deformed eggs would hatch, and detailed future studies on the possible blocking effect of GSK343 on egg development are warranted.

It is noteworthy that in the parasites that have been exposed to GSK343 a number of downregulated genes known to be related to the tegument do correlate with the phenotypic alterations detected by electron microscopy. Thus, dysferlin, which belongs to a family of genes similar to *C*. *elegans* ferlin and plays an important role in muscle fiber membrane repair [73] was found downregulated in males and could correlate with tegument surface damage. Also, sodium coupled neutral amino acid transporter, found downregulated in males, is known to influence the cell content of most amino acids, thus determining the overall size and the composition of the intracellular amino acid pool [74]. As amino acids represent a large fraction of cell organic osmolytes, changes of sodium coupled neutral amino acid transporter activity are followed by modifications in both cell amino acids and cell volume [74]; a decrease in the expression of this gene caused by GSK343 could be related to the appearance of bubbles seen in the male tegument. In addition, the expression downregulation of aquaporin 9 and of a number of ion transporters, observed in the GSK343-exposed parasites, probably resulted in a dysregulation of fluid, small solutes and ions homeostasis and may lie behind the distended appearance of the subtegumental region and the presence of bubbles in the tegument.

Treatment of adult worms and schistosomula with GSK343 was accompanied by a reduction in the H3K27me3 repressive mark, and by the upregulation of expression of hundreds of genes, as expected from a direct effect of an inhibitor of the EZH2 histone modifying enzyme (the enzyme that places the H3K27me3 repressive mark). In this respect, it should be noted that in females, males and schistosomula, some expression variability was observed among the control sample replicates as well as among the treated sample replicates, and that this is not unusual when dealing with non-clonal whole organisms. Nevertheless, a closer inspection of the expression heatmaps of Figs 8 and 9 shows that even when one replicate is more discordant from the others, most of those discordant genes have shown a change in expression (compared with their corresponding controls) that goes in the same direction as the change detected in the other replicates (albeit with a lesser magnitude of change).

Interestingly, downregulation in GSK343-treated females of tens of genes involved in DNA replication and ncRNA metabolism was observed, indicating that this was an indirect effect of epigenetic marks reprograming eventually related to an increase in expression of a transcriptional co-repressor [75] induced by the treatment.

Recent studies have shown that, as in vertebrates, *S*. *mansoni* also has a vast network of epigenetic marks [68] that could act in a stage-specific way on transcriptional control, resulting in different profiles of gene expression throughout its life cycle [15,76–78]. The present results suggest that DNA replication and ncRNA metabolism are two important processes related to egg-laying whose gene expression undergoes significant control by histone modifications. This is in addition to the known metabolic processes that control schistosome egg production such as fatty acid oxidation and the oxidative phosphorylation pathway [79], which may be required for fueling the energy required for replication and protein synthesis. Other egg-laying epigenetic regulators have recently been described, such as genomic DNA cytosine methylation [77], and two histone acetyltransferase modifying enzymes, SmGCN5 and SmCBP1 [57]. At this point we cannot identify the detailed mechanisms involved in expression downregulation of genes involved in DNA replication and ncRNA metabolism, which should be the subject of future studies; we speculate that such regulators could involve sex-biased microRNAs [78,80] and microRNAs involved in the regulation of ovary development [81].

The histone modifying enzymes are the central axes of the epigenetic regulation in eukaryotes, being involved in different signaling pathways. Our work points to *S*. *mansoni* EZH2 as a potential target for development of new schistosomicidal drugs. One concern is the ability to develop inhibitors that are active on the parasite and not on the human enzyme. In this respect, the parasite may be uniquely susceptible to a particular inhibitor, because schistosomes have only one H3K27 methyltransferase [12] whereas humans have two, namely EZH1 and EZH2 [82]. This difference suggests that the human enzymes may have undergone some particular evolutionary changes with respect to the single EZH2 in the parasite. Further work is required to screen existing compound libraries for selective inhibitors and/or to develop selective inhibitors based on structural analyses.

## Supporting information

S1 Text **This file contains the following three supplementary figures: Fig A. Common set of differentially expressed genes in GSK343-treated S. mansoni adult worms and schistosomula**. Venn diagrams of the number of genes that were detected as differentially expressed in the intersection of the gene output lists of the three statistical analysis pipelines that have been used (tool names are indicated next to each overlapping circle) in (i) adult females: 585 downregulated genes (left panel), and 447 upregulated genes (right panel) and in (ii) males: 567 downregulated genes (left panel), and 543 upregulated genes (right panel). The numbers inside the orange circles represent the genes affected in common between females and males: 45 downregulated genes and 82 upregulated genes. (iii) Venn diagrams of the number of genes that were detected as differentially expressed in the intersection of the three statistical analysis pipelines in schistosomula: 143 downregulated genes (left panel) and 29 upregulated genes (right panel). **Fig B. Validation by RT-qPCR of genes detected by RNA-seq as downregulated in females and schistosomula**. Expression of sixteen selected genes that are indicated in the x-axis was measured by the RT-qPCR method in RNA samples extracted from (i) female or (ii) schistosomula parasites exposed for 48 h in vitro either to vehicle (control, black bars) or to 20 μM GSK343 (treated, gray bars). Expression was normalized as indicated in the Methods, and the lowest normalized value among the control biological replicates was chosen as reference and arbitrarily set to 1. Relative expression of all other control and treated samples was calculated in relation to that value. Graphs show the mean ± S.D. of three biological replicates for each condition in females and of four replicates in schistosomula. Statistical significance was evaluated with the t-test and significant changes are marked with asterisk (p-value < 0.05). **Fig C. Heatmap of proteins detected by mass spectrometry as differentially abundant in S. mansoni adult worms treated with GSK343**. The heatmap shows the hierarchical clustering of 58 differentially abundant proteins (lines) detected in three biological replicates (columns) of adult worm total protein extract samples, either for controls or for treated parasites, as indicated at the top of the heatmap. Parasites were exposed for 48 h in vitro to vehicle (control) or to 20 μM GSK343. Protein abundance levels were measured by label-free quantitative (LFQ) mass spectrometry-based proteomics and the LFQ intensities are shown as Z-scores, which are the number of standard deviations below (blue) or above (yellow) the mean LFQ intensity value among treated and control samples for each protein; the LFQ intensity value Z-scores are color-coded as indicated on the scale at the bottom.
(PDF)

S1 Table List of primers used in qPCR. (XLSX)

S2 Table List of Smp genes downregulated in female adult worms treated with GKS343, along with their significant differential expression values from the three different statistical analyzes pipelines used in this study. (XLSX)

S3 Table List of Smp genes upregulated in female adult worms treated with GKS343, along with their significant differential expression values from the three different statistical analyzes pipelines used in this study. (XLSX)

S4 Table List of Smp genes downregulated in male adult worms treated with GKS343, along with their significant differential expression values from the three different statistical analyzes pipelines used in this study. (XLSX)

S5 Table List of Smp genes upregulated in male adult worms treated with GKS343, along with their significant differential expression values from the three different statistical analyzes pipelines used in this study. (XLSX)

S6 Table List of Smp genes downregulated in schistosomula treated with GKS343, along with their significant differential expression values from the three different statistical analyzes pipelines used in this study. (XLSX)

S7 Table List of Smp genes upregulated in schistosomula treated with GKS343, along with their significant differential expression values from the three different statistical analyzes pipelines used in this study. (XLSX)

S8 Table List of downregulated genes related with DNA replication, ncRNA metabolism and egg formation. (XLSX)

S9 Table Gut and tegument genes affected by GSK343. (XLSX)

S10 Table Proteins identified by proteomics. (XLSX)

S11 Table Proteins detected by mass spectrometry as differentially abundant in *S*. *mansoni* adult worms. (XLSX)

S12 Table Proteins uniquely detected in at least two replicates of one condition, either control or treated parasites, and in none of the other conditions. (XLSX)
