# Supplementary material for: Inhibition of histone methyltransferase EZH2 in Schistosoma mansoni in vitro by GSK343 reduces egg laying and decreases the expression of genes implicated in DNA replication and noncoding RNA metabolism
Source: PLoS Negl Trop Dis. 2018 Oct 26;12(10):e0006873. doi: 10.1371/journal.pntd.0006873 (PMC6221359; doi:10.1371/journal.pntd.0006873)
Supplement: S1 Text — This file contains the following three supplementary figures: Fig A. Common set of differentially expressed genes in GSK343-treated S. mansoni adult worms and schistosomula. Venn diagrams of the number of genes that were detected as differentially expressed in the intersection of the gene output lists of the three statistical analysis pipelines that have been used (tool names are indicated next to each overlapping circle) in (i) adult females: 585 downregulated genes (left panel), and 447 upregulated genes (right panel) and in (ii) males: 567 downregulated genes (left panel), and 543 upregulated genes (right panel). The numbers inside the orange circles represent the genes affected in common between females and males: 45 downregulated genes and 82 upregulated genes. (iii) Venn diagrams of the number of genes that were detected as differentially expressed in the intersection of the three statistical analysis pipelines in schistosomula: 143 downregulated genes (left panel) and 29 upregulated genes (right panel). Fig B. Validation by RT-qPCR of genes detected by RNA-seq as downregulated in females and schistosomula. Expression of sixteen selected genes that are indicated in the x-axis was measured by the RT-qPCR method in RNA samples extracted from (i) female or (ii) schistosomula parasites exposed for 48 h in vitro either to vehicle (control, black bars) or to 20 μM GSK343 (treated, gray bars). Expression was normalized as indicated in the Methods, and the lowest normalized value among the control biological replicates was chosen as reference and arbitrarily set to 1. Relative expression of all other control and treated samples was calculated in relation to that value. Graphs show the mean ± S.D. of three biological replicates for each condition in females and of four replicates in schistosomula. Statistical significance was evaluated with the t-test and significant changes are marked with asterisk (p-value < 0.05). Fig C. Heatmap of proteins detected by mass spectrom [file pntd.0006873.s001.pdf]

Supporting Information for

Inhibition of histone methyltransferase EZH2 in *Schistosoma mansoni in vitro* by GSK343 reduces egg laying and decreases the expression of genes implicated in DNA replication and noncoding RNA metabolism

Adriana S.A. Pereira[1,2], Murilo S. Amaral[1], Elton J. R. Vasconcelos[1,2], David S. Pires[1], Huma Asif[1], Lucas F. daSilva[1,2], David A. Morales-Vicente[1,2], Vitor C. Carneiro[3], Giuseppe Palmisano[4], Claudia B. Angeli[4], Marcelo R. Fantappie[3], Raymond J. Pierce[,5], João C. Setubal[2], Sergio Verjovski-Almeida[1,2*]

[1]Instituto Butantan, São Paulo, SP, Brasil;
[2]Departamento de Bioquímica, Instituto de Química, Universidade de São Paulo, São Paulo, SP, Brasil;
[3]Instituto de Bioquímica Médica Leopoldo de Meis, Universidade Federal do Rio de Janeiro, Rio de Janeiro, RJ, Brasil.
[4]Instituto de Ciências Biomédicas, Departamento de Parasitologia, Laboratório de Glicoproteômica, Universidade de São Paulo, São Paulo, SP, Brasil.
[5]Centre d'Infection et d'Immunité de Lille, CNRS UMR 8204, Inserm U1019, CHU Lille, Institut Pasteur de Lille, Université de Lille, Lille, France

*Corresponding author e- mail: verjo@iq.usp.b

S1 Text. This file contains the following three supplementary figures:

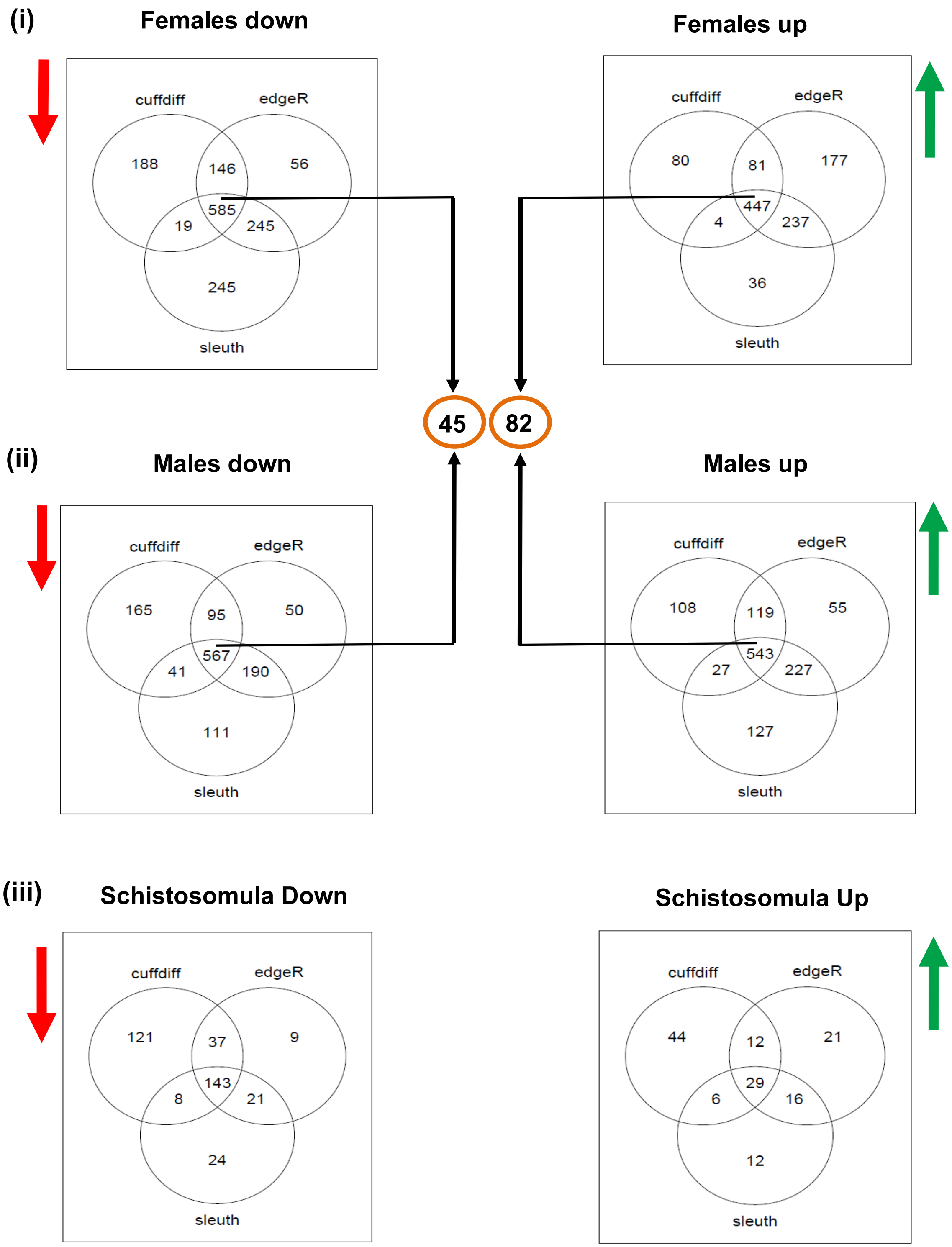

Fig A: Common set of differentially expressed genes in GSK343-treated *S. mansoni* adult worms and schistosomula. Venn diagrams of the number of genes that were detected as differentially expressed in the intersection of the gene output lists of the three statistical analysis tools that have been used (tool names are indicated next to each overlapping circle) in **(i)** adult females: 585 downregulated genes (left panel), and 447 upregulated genes (right panel) and in (**ii**) males: 567 downregulated genes (left panel), and 543 upregulated genes (right panel). The numbers inside the orange circles represent the genes affected in common between females and males: 45 downregulated genes and 82 upregulated genes. **(iii)** Venn diagrams of the number of genes that were detected as differentially expressed in the intersection of the three tools in schistosomula: 143 downregulated genes (left panel) and 29 upregulated genes (right panel).

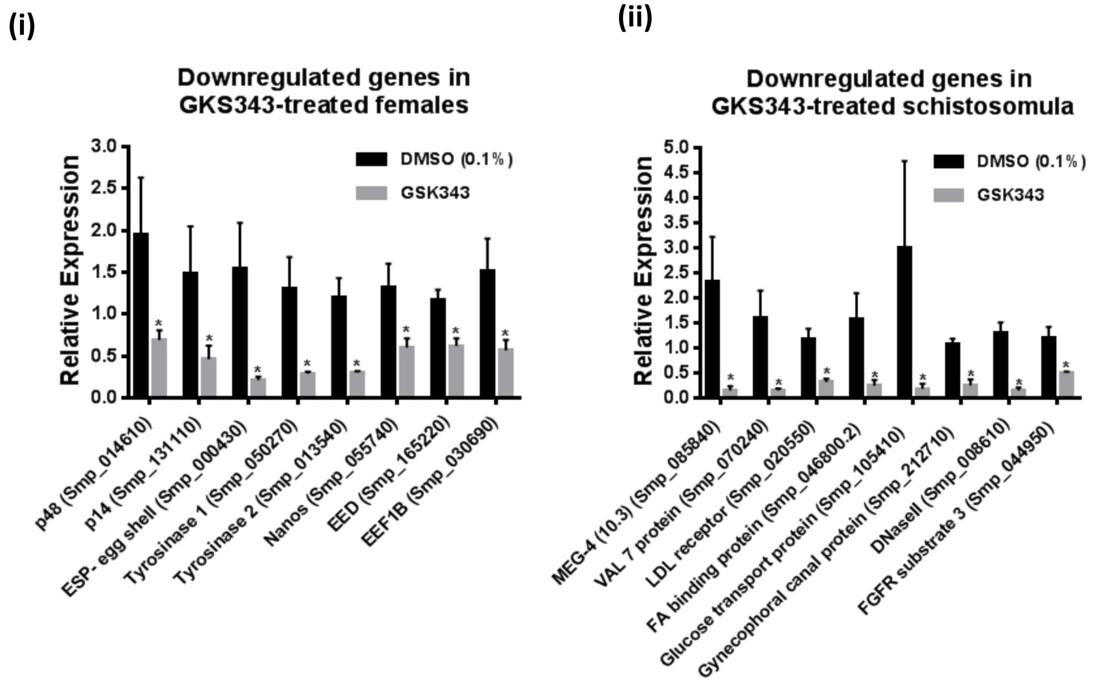

Fig B. Validation by RT-qPCR of genes detected by RNA-seq as downregulated in females and schistosomula. Expression of sixteen selected genes that are indicated in the x-axis was measured by the RT-qPCR method in RNA samples extracted from **(i)** female or **(ii)** schistosomula parasites exposed for 48 h *in vitro* either to vehicle (control, black bars) or to 20 µM GSK343 (treated, gray bars). Expression was normalized as indicated in the Methods, and the lowest normalized value among the control biological replicates was chosen as reference and arbitrarily set to 1. Relative expression of all other control and treated samples was calculated in relation to that value. Graphs show the mean ± S.D. of three biological replicates for each condition in females and of four replicates in schistosomula. Statistical significance was evaluated with the t-test and significant changes are marked with asterisk (p-value < 0.05).

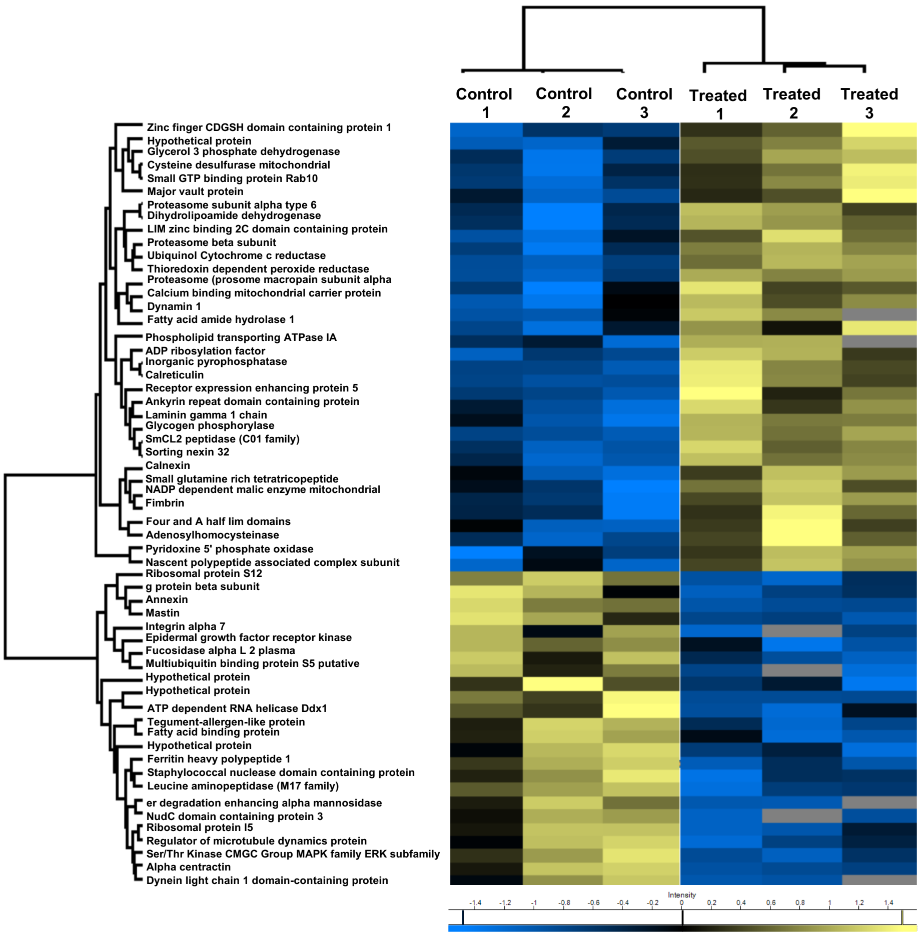

Fig C. Heatmap of proteins detected by mass spectrometry as differentially abundant in *S. mansoni* adult worms treated with GSK343. The heatmap shows the hierarchical clustering of 58 differentially abundant proteins (lines) detected in three biological replicates (columns) of adult worm total protein extract samples, either for controls or for treated parasites, as indicated at the top of the heatmap. Parasites were exposed for 48 h *in vitro* to vehicle (control) or to 20 μM GSK343. Protein abundance levels were measured by label-free quantitative (LFQ) mass spectrometry-based proteomics and the LFQ intensities are shown as Z-scores, which are the number of standard deviations below (blue) or above (yellow) the mean LFQ intensity value among treated and control samples for each protein; the LFQ intensity value Z-scores are color-coded as indicated on the scale at the bottom.
